# Virtual screening of compounds for the development of thyroid hormone analogues for potential application in cardiac regeneration

**DOI:** 10.1007/s10822-026-00787-5

**Published:** 2026-03-23

**Authors:** José de Anchieta de Oliveira Filho, Elton José Ferreira Chaves, Pedro Geraldo Pascutti, Enéas Ricardo de Morais Gomes

**Affiliations:** 1 Department of Biotechnology, Federal University of Paraiba, João Pessoa, Brazil; 2 Institute of Biophysics Carlos Chagas Filho, Federal University of Rio de Janeiro, Rio de Janeiro, Brazil

**Keywords:** Molecular docking, Infarction, Cardiac Injury, Cardiac regeneration and proliferation

## Abstract

Cardiovascular diseases remain the leading global cause of mortality, largely due to the limited regenerative capacity of adult cardiac tissue. Thyroid hormones, particularly tri-iodothyronine (T3), have been shown to stimulate cardiomyocyte proliferation through activation of the thyroid hormone receptor alpha (TRα), making this receptor a promising therapeutic target. Here we report a hierarchical and consensus, multi-level virtual screening pipeline integrating Molecular Mechanics (MM), Quantum Mechanics (QM), hybrid QM/MM calculations, and reactivity analysis based on the Fukui function to identify novel TRα agonists. Starting from 412 million compounds in the ZINC15 database, physicochemical filtering, validated pharmacophore matching, and docking guided by ROC curve optimization yielded 568 candidates, from which eight compounds were selected through chemically guided visual inspection. Binding affinity was evaluated with MD/MM-PBSA, PM7, and QM/MM (B3LYP/6-31G*/CHARMM36), and complemented by Fukui reactivity mapping to rationalize protein–ligand recognition. Two ligands, including the approved drug Cetraxate, consistently showed favorable interaction energies and reactivity patterns comparable to T3, suggesting agonistic potential. This work provides a rigorous, multi-scale computational framework and identifies two mechanistically supported TRα agonist candidates for future experimental validation in cardiac regeneration.

**Supplementary Information:**

The online version contains supplementary material available at 10.1007/s10822-026-00787-5.

## Introduction

Cardiovascular diseases (CVDs) consist of diseases affecting the heart (ischemic heart dysfunction, cardiomyopathy, congestive heart failure) and blood vessels (coronary artery disease, hypertension, and atherosclerosis) (1). With regards to ischemic heart disease, such as acute myocardial infarction (MI), a great loss of cardiac muscle is involved, compromising partial or total organ functionality, which may lead to death (2). In 2017 only, around 17.8 million cardiovascular disease-related deaths occurred worldwide, more than three-quarters of which were in low- and middle-income countries (3). Particularly, in 2019, data indicates that ischemic CVDs were responsible for 8.9 million deaths around the world (4). In 2021, approximately 9.5 million deaths related to ischemic heart disease occurred worldwide (5). Additionally, a higher number of deaths related to cardiovascular diseases have been observed during the coronavirus-19 (COVID-19) pandemic, both ‘directly’, through infection, and ‘indirectly’, through changes in healthcare, as well as decreased supply and demand for CVD treatment services (6).

Events that lead to cardiac cell death, especially cardiomyocytes, can lead to heart failure (HF), which is one of the main causes of mortality and morbidity. The main obstacle to functional recovery of the heart is the proliferative limitation of the cardiac muscle, in which instead of replacing the injured cells with cells with equivalent function, the response to injury is fibrotic scarring and hypertrophic remodelling (7, 8). The strategies of cardiac muscle cell regeneration and proliferation have great relevance and therapeutic potential. Having been studied for more than a century, a great deal of controversy has been generated about the regenerative capacity of the heart. However, postnatal regeneration of cardiac cells has been observed in animal models and even in humans (9). Thus, it is increasingly sought to understand the regenerative mechanisms of the cells in question, with great interest in developing approaches for clinical use of this regenerative and proliferative potential (10).

In this context, due to the known action of thyroid hormone (TH) in boosting embryonic development through its actions on cell proliferation and differentiation, the hypothesis was raised that it could influence aspects of tissue repair in cardiac cells. There is already preclinical and clinical evidence demonstrating its regenerative, repair and protective capacity for the heart (11, 12). Additionally, it has been reported that regenerative effects of TH treatment over the myocardium occur via the thyroid alpha 1 receptor (TRα), being it a promising target for the development of new drugs aimed at post-ischemic heart injury treatment (13).

Computational methodologies are of great importance for the discovery and optimization of new drugs, and can make the process more efficient, safer, and more cost-effective (14). Currently, computer simulation techniques are just as important for the study of biological systems as experimental methods, usually acting in together. In particular, it has become possible to study the electronic structure of biological macromolecules with thousands of atoms and derive useful information for understanding protein-ligand interaction due to the development of semiempirical methods (15), computational availability, and robust electronic structure analysis tools (16, 17).

Among these, virtual screening strategies, particularly structure-based methods such as molecular docking, allow the prediction of ligand binding modes and affinities by evaluating the complementarity between candidate molecules and the target binding site. However, conventional docking approaches often rely on simplified scoring functions, which may limit their accuracy in capturing complex physicochemical interactions. To overcome these limitations, hierarchical virtual screening pipelines have been increasingly employed, combining multiple levels of theory to improve prediction reliability. In such approaches, initial filtering and docking are followed by more refined calculations, including molecular dynamics (MD) simulations, free energy estimations, and quantum mechanical (QM) methods, enabling a more detailed description of protein–ligand interactions.

In addition to energetic evaluation, electronic structure descriptors have been explored to provide further insight into molecular recognition processes. The Fukui function, derived from conceptual density functional theory, describes the susceptibility of atomic sites to electrophilic and nucleophilic attacks based on frontier molecular orbitals (18). Although traditionally applied to chemical reactivity, recent studies have demonstrated its potential in analyzing intermolecular interactions and charge transfer effects in biomolecular systems (16, 17). The relationship between Fukui’s relativity and the biological activity of agonist molecules (capable of activating/promote biological function) is still little explored, especially in complex with proteins as receptors. In this sense, reactivity is used in this work considering intermolecular interactions through charge transfer coupling, even though chemical reactions do not occur as in enzymes.

In light of the problems surrounding the treatment of cardiac muscle lesions, particularly a shortage of drugs for this purpose, and in light of the potential of THs in the treatment of these lesions, the development of new drugs is necessary, wherein the TH might be promising models for this approach. Here, using methodologies based on Molecular Mechanics (MM) and Quantum Mechanics (QM), we explore the potential of the Fukui function for predicting intermolecular interactions in a hierarchical virtual screening in search of TRα agonist molecules with characteristics of drugs of clinical interest, aiming to develop therapeutic compounds for the treatment of lesions to the myocardium.

## Methods

Aiming to find potentially agonist compounds for TRα, a hierarchical virtual screening was performed, using the commercial molecule database ZINC 15 (19) (details in 2.1 Obtaining the Database). For this, initially the library of compounds was inspected with the software FILTER version 3.1.0.3 (details in 2.3 Physical-Chemical Filter) (20) selecting those with physicochemical characteristics similar to orally absorbable drugs. Subsequently, a pharmacophore filter was applied using the ROCS version 3.3.0.3 (details in 2.4 Pharmacophore Screening) (21). Then, through the statistical approach of the ROC curve a cut-off point was established, rescuing the most promising candidate compounds. Next, the molecules selected in the previous step were classified by the OEDocking FRED version 3.3.0.3 (details in 2.5 Molecular Docking) (22).

**Fig. 1:**
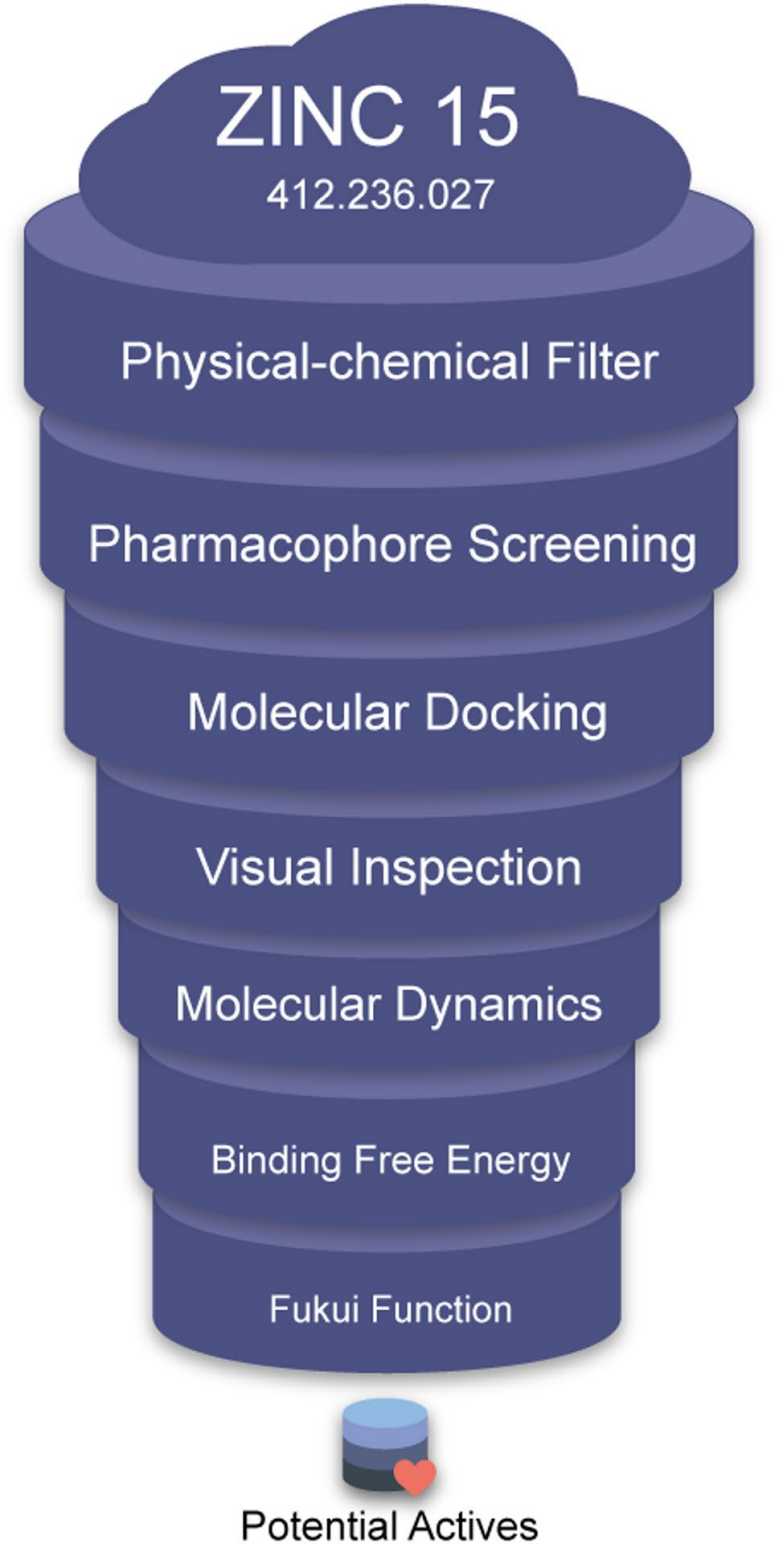
Schematic summary of the virtual screening flow for TRα agonists. A total of 412,236,027 compounds were initially retrieved from the ZINC15 database. After applying physicochemical filters, 1,798,667 compounds remained. Pharmacophore screening reduced this set to 10,633 compounds, which were subjected to molecular docking, yielding 568 candidates. Visual inspection selected 8 compounds, which were further evaluated by molecular dynamics simulations (8 complexes maintained). Subsequent binding free energy calculations and Fukui function analyses were performed on these 8 compounds, resulting in 2 final candidates identified as potential TRα agonists

With the ligands selected by molecular docking, a visual inspection step was performed, aided by the report generated through the Docking Report program (details in 2.6 Visual Inspection of the Candidates) (21). Continuing the screening, Molecular Dynamics simulations were performed with NAMD program (23) and Free Energy of Binding by Molecular Mechanics/Poisson–Boltzmann Surface Area (MM/PBSA) were calculated with AMBER program package (24) (details in Sect.  2.7 and 2.8), semiempirical Quantum Mechanics, PM7 (15), and hybrid Quantum Mechanics Molecular Mechanics (QMMM) (B3LYP 6-31G*/CHARMM36) were calculated with Orca 5 software (25) (details in 2.9 Quantum Mechanics Molecular Mechanics Hybrid Calculation). Additionally, the Fukui reactivity descriptor was calculated to understand the main interactions involved in the formation of the complex (26) (details in 2.10 Reactivity Descriptor Calculations). Finally, the ligands with the highest agonist potential for TRα were obtained. The screening flow is summarized in Fig. 1.

### Obtaining the database

Candidate molecules were obtained from the ZINC 15 commercial molecule database (19) via the web platform and used for screening those that met the following characteristics: (a) 3D representation; (b) standard reactivity; (c) available in stock; (d) pH 7.4; (e) uncharged; and (f) classified as lead-like.

### Validation of computational methods

Validation of the computational tests was performed using active and inactive molecules for TRα. The molecules were obtained using ChEMBL25 as a base by searching for the term “*Thyroid hormone receptor alpha*”, targeted with code CHEMBL1860, including molecules that showed agonist, antagonist, and no activity in single protein and cell culture assays. Thus, these data were grouped into two databases for validation: (1) a database with active agonist compounds (Table S2); and (2) a database with inactive ligands, including: antagonists to the target and decoys, assembled by the DUD-E platform (27), which generates molecules with chemical characteristics similar to the actives, however without 3D similarity, with the inactive database being the summation of antagonists and decoys from DUD-E.

Receiver Operating Characteristic (ROC) curve analysis was used to evaluate and optimize the performance of the virtual screening methods, as well as to define cut-off points to retrieve the molecules with the highest potential activity. Graph construction and analysis were performed by the EasyROC platform (28), which were used to evaluate the Area Under the Curve (AUC) and scatter plots as described by Triballeau et al. (2005) (29).

### Physical-chemical filter

To select candidates that exhibit good absorption, distribution, metabolism and excretion, FILTER version 3.1.0 was used.3 from Open Eye^®^, preserving candidates that met the inclusion criteria: (a) molecular mass of less than 450 daltons; (b) n-octanol/water partition logarithm less than 4.5 and greater than − 3.5; (c) less than 4 rings; (d) less than 10 non-terminal single bonds; and (e) less than 5 hydrogen bond donors and no more than 8 hydrogen bond acceptors; based on the study by Oprea et al. (2001) (20).

### Pharmacophore screening

ROCS software of chemical similarity analysis is a well-established strategy in drug discovery, enabling the identification of compounds with related biological activity based on structural resemblance. While two-dimensional (2D) methods rely on molecular topology, three-dimensional (3D) approaches incorporate conformational flexibility and spatial arrangement, allowing the identification of compounds with similar shape and physicochemical properties, even when their chemical structures are not intuitively related. In this context, ROCS employs a 3D superposition methodology specifically designed for large-scale virtual screening. The method aligns molecules through rigid-body optimization, maximizing their volumetric overlap using a Gaussian-based representation of atomic volumes, which provides a smooth and computationally efficient approximation of molecular shape. This approach enables the identification of structurally diverse compounds that share similar steric and chemical features with a reference ligand, making it particularly suitable for exploring large chemical libraries in drug discovery pipelines (21).

To quantify molecular similarity, ROCS employs overlap-based metrics derived from both shape and chemical feature representations, using similarity coefficients such as Tanimoto and Tversky. These measures are based on three fundamental terms: the self-overlap of each molecule (selfA and selfB) and the overlap between them (overlap_AB_). The Tanimoto coefficient is a symmetric measure defined as the ratio between the shared overlap and the total volume of both molecules, yielding values between 0 and 1, where higher values indicate greater similarity. In contrast, the Tversky coefficient is an asymmetric metric that introduces weighting factors (α and β) to bias the comparison toward a reference molecule, allowing greater emphasis on specific structural features. This property makes Tversky particularly useful in virtual screening, as it enables prioritization of compounds that better match the reference ligand, even when overall similarity is lower. Together, these metrics provide complementary perspectives on molecular resemblance, enhancing the identification of candidate molecules with relevant structural and chemical features. The expressions representing Tanimo and Tversky are.

1 $$\mathrm{Tan} imoto_{{A,B}} = \frac{{overlapAB}}{{selfA~ + ~selfB~ - ~overlapAB}}$$

2 $$Tversky_{{A,B}} = \frac{{overlapAB}}{{\alpha ~*~selfA~ + ~\beta ~*~selfB}}$$

In addition to shape-based alignment, ROCS incorporates chemical feature matching through the so-called “color” component, which accounts for the spatial distribution of key pharmacophoric features. In this approach, functional groups such as hydrogen bond donors and acceptors, charged groups (anions and cations), hydrophobic regions, and aromatic rings are represented as Gaussian functions centered on heavy atoms. Unlike shape Gaussians, color features are modeled using “hard” Gaussians with steep gradients, ensuring that matches occur only when features are closely aligned in space, thereby increasing specificity. The definition of these features is governed by predefined color force fields (e.g., Implicit Mills–Dean), which assign interaction types and weights based on chemical functionality. Color similarity can be evaluated independently (e.g., Color Tanimoto, Color Tversky) or combined with shape similarity (e.g., Tanimoto Combo, Tversky Combo), enabling a more comprehensive assessment of both steric and chemical complementarity between molecules during virtual screening.

In ROCS, molecular shape is defined as a three-dimensional scalar field describing the volume occupied by a molecule, where shape similarity is quantified based on the degree of overlap between such fields. Rather than using discrete hard-sphere representations, ROCS employs a continuous Gaussian-based description of atomic volumes, allowing efficient and smooth calculation of molecular overlap and alignment. In this framework, the similarity between two molecules is determined by maximizing the overlap between their Gaussian volume representations, which depends on both their self-overlaps and mutual overlap. This formulation enables the use of well-established similarity metrics, such as Tanimoto and Tversky coefficients, derived directly from these overlap terms. The Gaussian representation improves computational efficiency and avoids discontinuities associated with hard-sphere models, facilitating robust optimization and enabling accurate comparison of molecular shapes even for structurally diverse compounds.

Pharmacophore screening was performed using the ROCS software from Open Eye^®^ (21). The query model was generated by the visual interface, vROCS, considering all the features of the T3 ligand, considering the conformation obtained by X-ray crystallography, available in the Protein Data Bank (PDB) (30), with PDB ID 2H79 (31). All 13 functions available with ROCS software were validated, selecting those with the highest AUC and with the best population dispersion profile, considering the validation molecules. Then, the candidate molecules coming from the physicochemical filter screening step were evaluated with ROCS software (21), generating scores for all available functions, without ranking the candidates. The molecules were selected by applying a cutoff value defined through ROC curve analysis and supported by density distribution plots. The selected threshold corresponds to the point that maximizes the discrimination between active compounds and decoys, thereby defining the optimal balance between sensitivity and specificity.

### Molecular docking

Molecular docking was performed with the OEDocking software FRED, using the semi-flexible docking approach, keeping the receptor rigid and the ligand flexible. The low energy conformations of the molecules were generated by the OMEGA software (32). Docking was performed using the OEDocking suite FRED (OpenEye Scientific Software) with a semi-flexible approach: the receptor was kept rigid while the ligands were treated as flexible. A conformer ensemble for each ligand was first generated with OMEGA, employing the Merck molecular force field (MMFF94) (33) for conformer sampling and optimization (32).

The OEDocking software FRED uses a precomputed negative image of the receptor binding site and performs an exhaustive, deterministic shape-based matching of ligand conformations within this volume. In this context, sampling is controlled by parameters such as translational and rotational step sizes, which define the granularity of ligand placement and orientation during docking. In the present study, docking was carried out using standard resolution settings, corresponding to a translational step size at resolution level 1 and a rotational step size of 1.5 Å, ensuring a fine and systematic exploration of ligand poses within the binding cavity (32).

The structure with PDB ID 4LNW was used to perform the docking simulation (Figure S5). In order to optimize molecular docking performance, some restrictions based on the literature were defined. The hydrogen bond with the hydroxyl group of the phenol group of T3 is fundamental for the agonist activity of TRα (34). Interactions with the carboxyl group of the hormone are mediated by three arginine residues 228, 262, and 266. Additionally, the interaction between the amino group of T3 and Ser277 promotes selectivity between the α and β isoforms (35, 36). To define the best configurations, the following models were generated: (a) 4lnw_oti, with the external boundary parameters increased; (b) 4lnw_His381, interactions with His381 favored by a hydrogen donor; (c) 4lnw_Arg228, interactions with Arg228 favored by a hydrogen acceptor; and (d) 4lnw_His_Arg, with both aforementioned. To evaluate the molecular docking models and parameters, performance was assessed through ROC curve validation, comparing AUC values. The validation data were those described in Sect.  2.2 Validation of Computational Methods.

### Visual inspection of the candidates

The compounds selected in the molecular docking stage were subsequently evaluated through a guided visual inspection, using reports generated by OpenEye Docking Report^®^ and structural analysis of the ligand-receptor complexes. This step was performed to refine the selection based on well-established structural characteristics of TRα-ligand interactions.

To reduce subjectivity and ensure reproducibility, objective criteria were defined based on interaction patterns supported by the literature. It is known that hydrogen bonding involving the hydroxyl group of the phenolic portion of T3 is critical for TRα agonist activity (34). In addition, interactions between the carboxyl group of the ligand and arginine residues (Arg228, Arg262, and Arg266) play a key role in stabilizing the ligand within the binding pocket. Furthermore, interactions involving Ser277 have been reported to contribute to selectivity between the TRα and TRβ isoforms (34–36).

Based on these structural requirements, the following inclusion criteria were applied: (a) the presence of a phenol group or an equivalent hydrogen donor capable of interacting with His381; (b) the presence of a hydrogen bond acceptor that interacts with Arg228; and (c) stabilizing interactions with Ser277.

### Molecular dynamics simulations

Molecular Dynamics simulations were performed with three models: (a) Apo, only the protein unit of TRα, considered inactive; (b) Holo, TRα bound to T3, active; and (c) TRα bound to the candidate ligand. The system was assembled using the complex generated by Molecular Docking. Initially, the pk_a_ values of the amino acid residues were solved using PDB2PQR (37) considering pH 7.4. A cubic box with a 15 Å thick and hydration layer was assembled with the TIP3P water model and the total charges were equivalently neutralized with Na^+^ ions using the AMBER package (24). The ff14SB force field for proteins (38) was used, whereas the force field of the ligands were generated with Antechamber, also based on the amber force field with charge parameterization using semi-empirical calculations (39).

All simulations were performed using the NAMD software (23) under periodic boundary conditions. Simulations were conducted in the isothermal–isobaric (NPT) ensemble at 310 K and 1 atm. Temperature was controlled using a Langevin thermostat with a damping coefficient of 1 ps^−^¹, while pressure was maintained using a Langevin piston barostat. Long-range electrostatic interactions were treated using the Particle Mesh Ewald (PME) method with a grid spacing of 1.0 Å and a tolerance of 1.0 × 10^− 6^. A cutoff of 14 Å was applied for van der Waals interactions. Bonds involving hydrogen atoms were constrained, and the SETTLE algorithm was used for water molecules, allowing the use of a 2 fs integration timestep. The simulation protocol consisted of four stages: (1) energy minimization, initially with the protein fixed and subsequently with all atoms free; (2) a heating phase from 0 K to 310 K with gradual temperature increments of 0.01 K per step; (3) an equilibration phase of 4 ns at constant temperature and pressure; and (4) a production run of 10 ns under NPT conditions.

It is important to note that the molecular dynamics simulations performed in this study were primarily intended as a structural refinement step rather than for exhaustive conformational sampling. Short MD simulations have been shown to be effective in improving docking-derived binding modes and evaluating ligand stability through RMSD analysis, with limited gains observed when extending simulations to longer timescales (40). In this context, the 10 ns production time used here represents a balance between computational efficiency and structural relaxation, enabling the application of the protocol to large numbers of candidate molecules.

The resulting trajectories were analyzed by evaluating the Binding Free Energy by MM/PBSA, PM7 MOZYME and QMMM (B3LYP 6-31G*/CHARMM36) and Fukui’s reactivity descriptor.

### Free energy calculation using molecular mechanics/poisson boltzmann surface area method

The Molecular Mechanics Poisson-Boltzmann and Surface Area Continuum Solvation (MM/PBSA) method (41) was used to perform the ligand binding free energy calculations using the MMPBSA.py script from the Amber Tools package (24). The procedure was carried out using the trajectories resulting from the DM production simulations, considering frames from 1 to 5000 with an interval of 5, resulting in a total of 1000 frames used for energy estimation. The configuration parameters were adapted from the work of the group by Miller et al. (2012) (42), which describes optimization tests of the parameters of the MMPBSA.py script. The Poisson–Boltzmann implicit solvent model was applied with a solvent dielectric constant (ε_out_) of 80 and a solute dielectric constant (ε_in_) of 1.0. A salt concentration of 0.1 M was used (istrng = 0.100). The cavity surface tension and offset parameters were set to 0.0378 and − 0.5692, respectively, following standard recommendations. Additional parameters included a probe radius of 1.4 Å, fill ratio of 4, grid scale of 2.0, and linear solver iterations set to 1000.

### Quantum mechanics molecular mechanics hybrid calculation

To calculate the affinity of candidate ligands for TRα, we employed a hybrid Quantum Mechanics/Molecular Mechanics (QM/MM) approach, which allows the treatment of different regions of large biomolecular systems using distinct levels of theory. The QM region was defined as all residues within a radius of approximately 5 to 10 Å from the ligand, encompassing the entire binding cavity and key interacting residues. The QM region was calculated using the hybrid density functional B3LYP (43, 44), employing the 6-31G basis set with polarization functions on non-hydrogen atoms. A self-consistent field (SCF) convergence criterion of 1.0 × 10^−^⁸ a.u. was used for energy convergence. All QM calculations were performed using the ORCA 5 software (25). The interaction between the QM and MM regions was treated using an electrostatic embedding scheme, in which the MM point charges polarize the QM electron density. The MM region was described using the CHARMM36 force field (45).

The systems were prepared utilizing the final conformations obtained from the equilibration MD simulations. The binding energy was calculated following Eq. 3:

3 $$\Delta E~ = ~E_{{Tr\alpha - ligand}} - \left( {E_{{ligand}} + E_{{Tr\alpha }} } \right)$$

### Reactivity descriptor calculations

The Trα-ligand candidate complexes equilibrated with MD simulations were calculated using the semi-empirical PM7 method (Stewart, 2013), employing an implicit solvent with conductor-like screening model (COSMO) through the linear scaling algorithm MOZYME implemented in the MOPAC2016 program (15), as described in Rocha-Santos et al. (2021) (26). Subsequently, the PRIMoRDiA software (16) was utilized to calculate the Fukui function for each atomic center *k* of the system. This approach employs a convenient mathematical framework, computing the descriptor in two functions: the left Fukui function (f^−^) (Eq. 4), corresponding to susceptibility to electrophilic attack, the right Fukui function (f^+^) (Eq. 5), specific for susceptibility to nucleophilic attack, and the global effects f^0^ (Eq. 6).

4 $$f^{ - } \left( k \right)~ = ~\mathop \sum \limits_{{v \in k}}^{{AO}} \left| {C_{{vHOMO}} } \right|^{2} + \mathop \sum \limits_{{v \in k}}^{{AO}} \left| {C_{{vHOMO}} C_{{\mu HOMO}} } \right|S_{{\mu v}}$$

5 $$f^{ + } \left( k \right)~ = ~\mathop \sum \limits_{{v \in k}}^{{AO}} \left| {C_{{vLUMO}} } \right|^{2} + \mathop \sum \limits_{{v \in k}}^{{AO}} \left| {C_{{vLUMO}} C_{{\mu LUMO}} } \right|S_{{\mu v}}$$

6 $$f^{0} = \frac{{f^{ + } \left( k \right)~ + ~f^{ - } \left( k \right)}}{2}$$

The first term of the f^−^ function (Eq. 2) is the summation of the squared *v* coefficients of the atomic orbitals (AOs), associated with the HOMO (and the HOMO-n orbitals within the chosen range, 3 eV in this study), belonging to atom *k*. The second term is the summation of the product between the *C*_*v*_ and *C*_*µ*_ coefficients, multiplied by the corresponding elements of the overlap matrix *S*_*µv*_. In the second equation, f^+^, the same expression is used, applying the corresponding AOs to the LUMO (and the LUMO + n orbitals within the chosen range). Then, f^−^ and f^+^ are combined to understand the overall effects of the Fukui indices for each atom, f^0^ (Eq. 6) (46). Finally, we visually represent the reactivity indices with a color gradient, with red representing non-reactive regions of the protein-ligand complex, white neutral regions and blue reactive regions. The color gradient varies according to intensity, from non-reactive regions (intense red) to maximum reactive regions (intense blue).

## Results

Virtual Screening started from 412,236,027 million molecules deposited in the ZINC 15 database (19). Then, 2,291,411 molecules were obtained, based on the criteria defined in the methodology, to start selecting molecules with agonist potential for TRα, using the pipeline demonstrated in Fig. 1.

### Physical-chemical filter

The 2,291,411 molecules resulting from the initial selection performed in ZINC 15 were analyzed with FILTER, aiming to select compounds with pharmacological potentiality, according to the physicochemical standards described by Oprea et al. (2001) (20). Then, 792,744 compounds that did not meet the inclusion criteria were excluded obtaining 1,498,667 molecules with drug characteristics, used as input to the pharmacophore filter.

### Pharmacophore screening

**Fig. 2:**
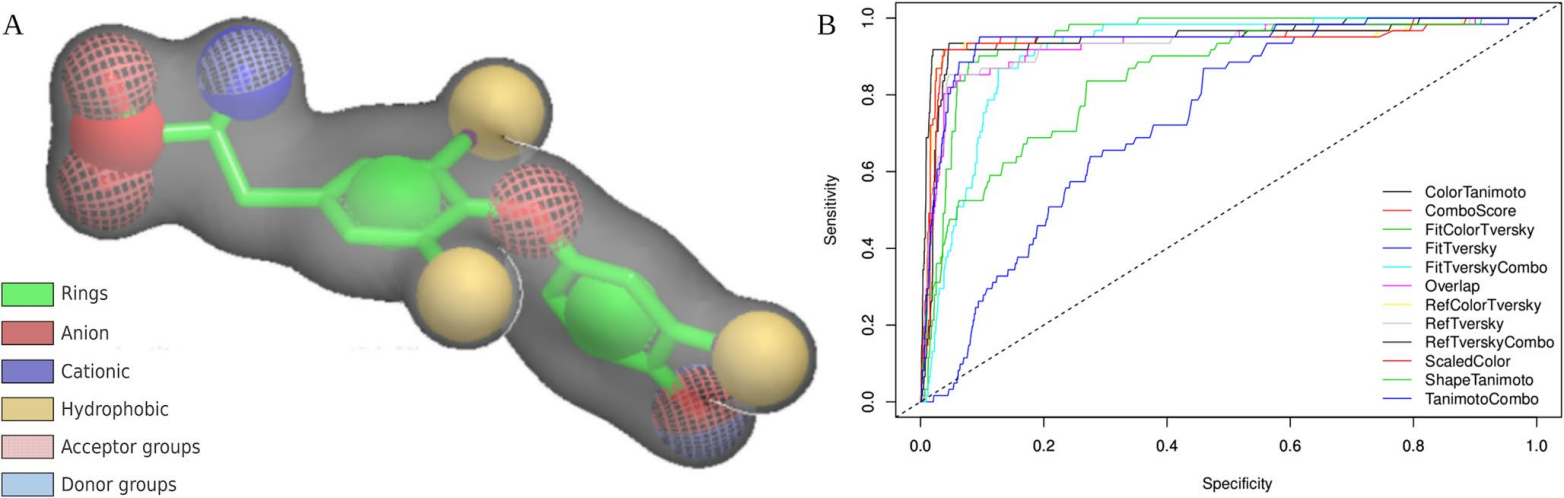
vROCS-generated pharmacophore model for T3. (**A**) Pharmacophore model generated from the T3 molecule with ROCS program. The spheres are representations of virtual atoms; and the gray space demonstrates the molecular volume. In solid green sphere, rings; in solid yellow, hydrophobic groups; in solid blue, cationic groups; in solid read, anionic groups; in mesh blue, hydrogen bonds donor groups; and in mesh red, hydrogen bonds acceptor groups. (**B**) Receiver Operating Characteristic (ROC) curves used to validate the 13 scoring functions applied in the pharmacophore model. The curves represent the ability of each function to discriminate between active and inactive compounds targeting TRα. Sensitivity is plotted against specificity, and the dashed diagonal line indicates random classification. Functions with curves closer to the upper-left corner demonstrate better performance and higher predictive power, as reflected by higher Area Under the Curve (AUC) values

The structure-based pharmacophore model was generated considering all chemical groups and the conformation of T3, depicted in Fig. 2. The model was validated with the database of active and inactive ligands. The results of the thirteen available ROCS scoring functions were analyzed by ROC curve, based on AUC (Figure S1) and scatter plots (Figure S2, functions selected in Table 1). Thus, an appropriate cutoff point was defined for each function, valuing the sensitivity of the test, i.e., using a score capable of retrieving the maximum of potentially active ligands, valuing the innovation in the molecules, even if a high number of false positives is obtained. The functions *Ref Tversky combo*,* Color Tanimoto*,* Combo Score*,* Ref Color Tversky*,* Scaled Color* and *Color Score* were selected to be used in the pharmacophore screening, as these presented AUC higher than 0.94 and a dispersion profile of the populations where it is possible to define a cut-off point separating the active from the inactive. A cut-off point was then defined for each function based on the ligand score density plots. Table 1 shows the AUC values and the defined cutoff point for the selected functions. Finally, the molecules obtained by the physicochemical filter were evaluated by ROCS, and 10,633 candidate molecules potentially agonistic for TRα were selected.

**Table 1:** Cut-off point and AUC of the ROCS scoring functions. Functions in bold were selected for use in the pharmacophore screening

Scoring Function	AUC	Cut-off
Ref Tversky Combo	0.95295	1.2
Color Tanimoto	0.94651	0.28
Fit Color Tversky	0.94598	-
Combo Score	0.94493	1.05
Tanimoto Combo	0.94361	-
Ref Color Tversky	0.9415	0.39
Scaled Color	0.94096	0.39
Color Score	0.9408078	-4.8
Ref Tversky	0.93198	-
Overlap	0.93044	-
Fit Tversky Combo	0.91272	-
Tanimoto Shape	0.84875	-
Fit Tversky	0.73226	-

### Molecular docking and visual inspection

All models with different docking configurations showed AUC greater than 0.89, as can be seen in Fig. 3 of the supplementary material. Then, an initial screening was performed with the molecules selected in the pharmacophore screening step with optimized model with lower AUC (4lnw_oti), using a less restrictive cut-off point, aiming to make the most of the number of potential actives, using a cut-off point of -11.62, selecting 7,208 molecules. Next, parallel docking was performed with the interaction-restricted 4lnw_his381 and 4lnw_his_arg molecules using cut points − 13 and − 10 respectively, with 568 molecules being obtained. Additionally, visual inspection was performed based on the inclusion criteria defined in the methodology, and 8 candidate ligands were selected as potential agonists for TRα.

**Fig. 3:**
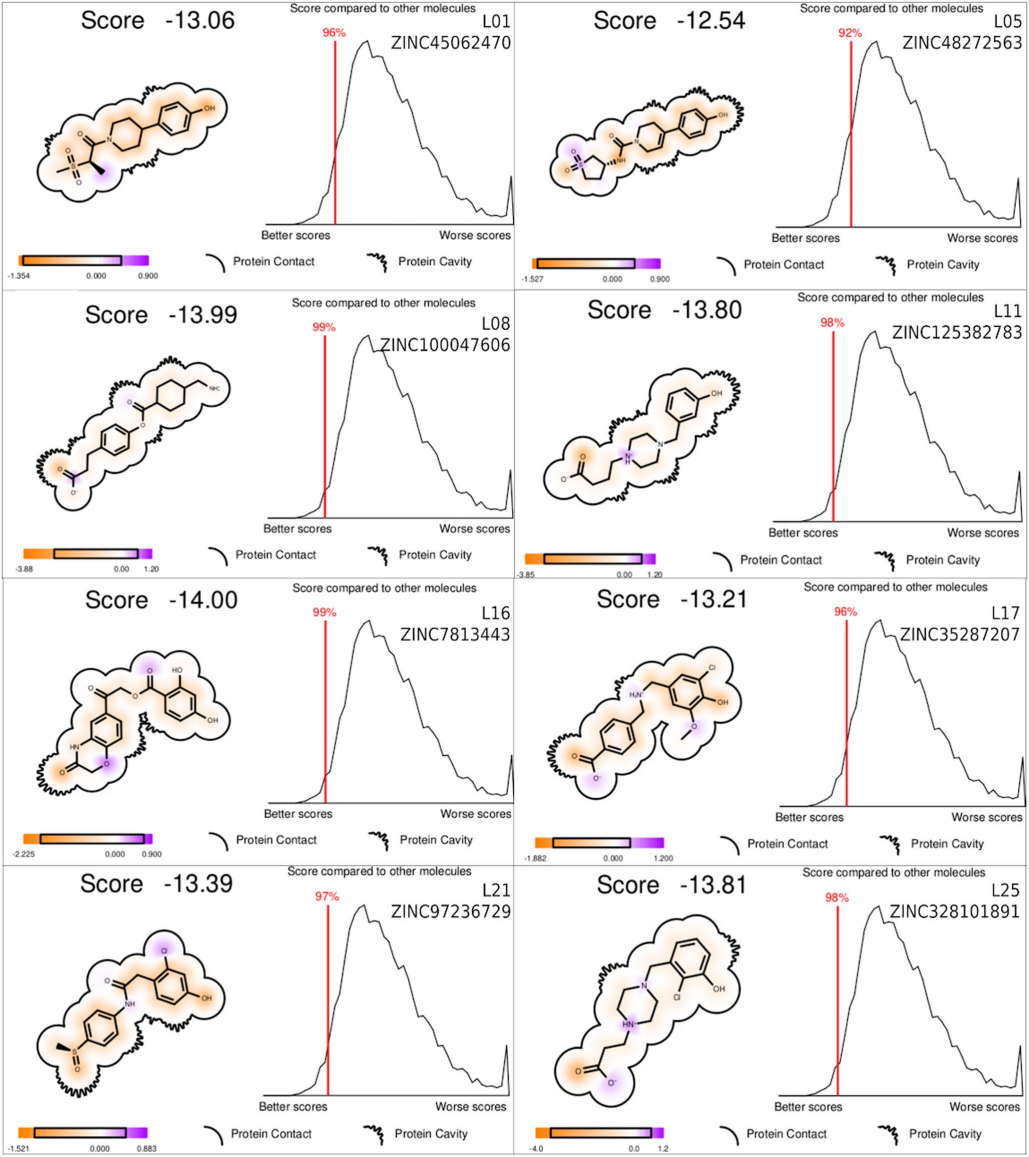
2D representation and score compared to other molecules molecules of the ligands selected in the molecular Docking and visual inspection. The individual scores of each molecule, score compared to other molecular docking candidates, 2D representation and the constituents of the score are represented in an orange and purple color scale. The orange-colored atoms contribute more effectively to a better score, while the purple coloring indicates penalties for the total docking score

### Binding energies

**Fig. 4:**
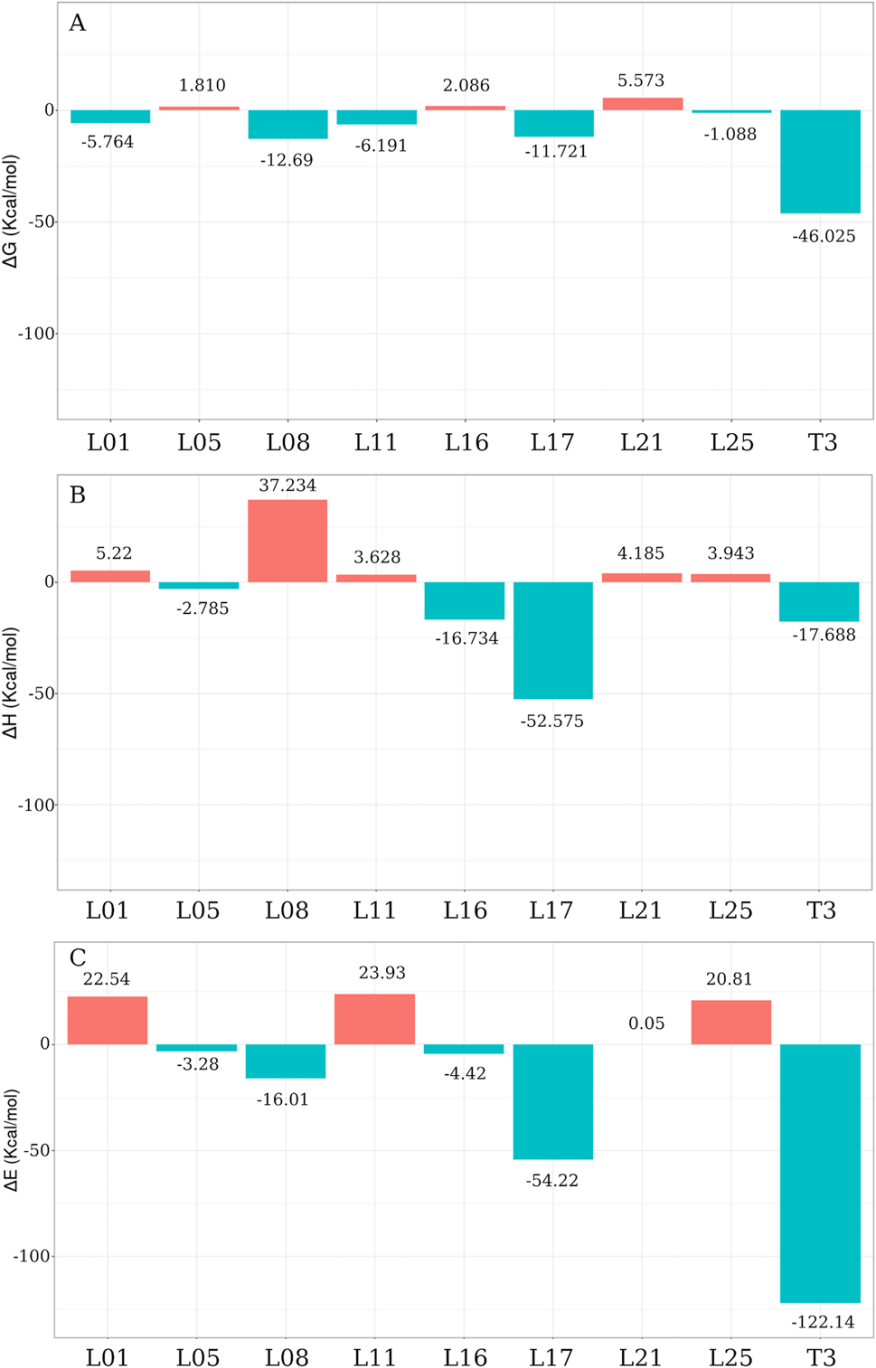
Binding energy, ∆G, ∆H and ∆E, of TRα complexes. DeltaH values calculated using multiple approaches, including (A) Molecular Mechanics/Poisson Boltzmann Surface Area (MM/PBSA), (B) the semiempirical quantum method PM7, and (C) the Quantum Mechanics/Molecular Mechanics (QMMM) B3LYP 6-31G*/CHARMM36, for both the reference ligand T3 and the candidate ligands targeting TRα agonism

To select the most promising candidate molecules, reranking was performed using binding energy calculations between the ligand and TRα. Subsequently, utilizing the structures obtained after MD simulations, we calculated ΔH Binding at different levels of theory, including classical mechanics (MM/PBSA), semiempirical quantum methods (PM7), and hybrid QMMM (B3LYP 6-31G*/CHARMM36). Figure 4 summarizes the Binding energy values for all candidate ligands and T3 at the different levels of theory.

The stability of the ligand binding modes was initially assessed through RMSD analysis of ligand heavy atoms the 10 ns molecular dynamics simulations (Figure S6). In general, the systems exhibit rapid equilibration within the first nanoseconds, followed by relatively stable RMSD profiles, indicating that the docking-derived poses remain preserved within the binding site. Most ligands show fluctuations around 2–3 Å, consistent with stable binding conformations, while a few compounds display higher variability, suggesting greater flexibility within the pocket. These results support that the MD simulations were sufficient to achieve local structural relaxation of the complexes, providing reliable conformations for subsequent binding energy calculations.

Divergences in energy values were observed between the MM/PBSA and the quantum methods (PM7 and QMMM), particularly in the calculated values for T3, which were − 46.0253 kcal/mol, -17.68817 kcal/mol, and − 122.14 kcal/mol for MM/PBSA, PM7, and QMMM B3LYP 6-31G*, respectively. On the other hand, quantum methods presented consistent and similar values. However, despite the distinct energy values obtained from the theoretical approaches, candidate ligands L08 and L17 exhibited favorable energy values for complex formation with the MM/PBSA and DFT methods. Therefore, these candidate molecules are considered potential TRα agonists.

### Protein-ligand interaction with fukui function

**Fig. 5:**
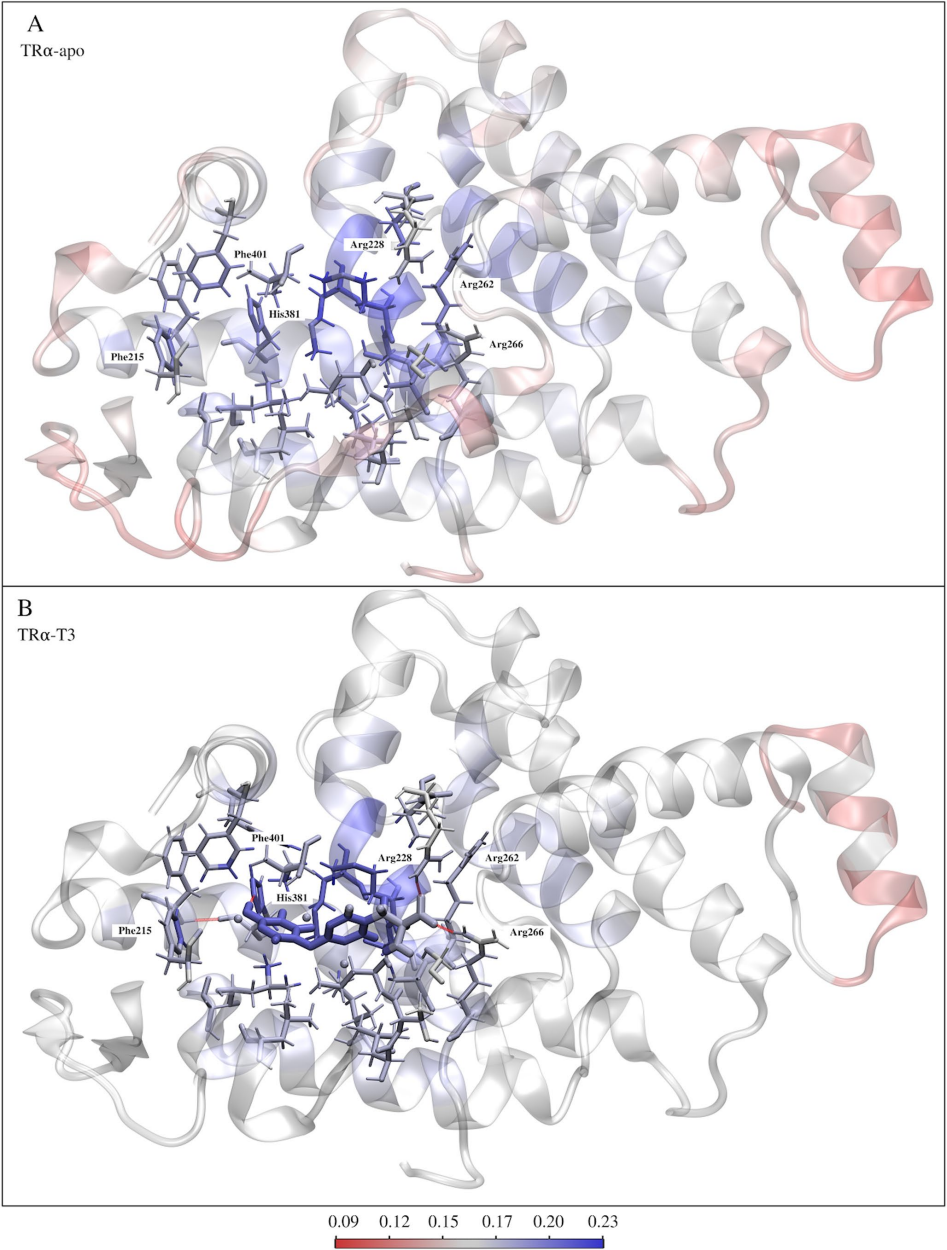
Representation of Fukui reactivity descriptor for TRα complexes with (A) TR-apo and (B) T3. The reactivity index is colored with a red-white-blue color gradient, red being less reactive and blue being more reactive, as per the color legend. The T3 hormone is highlighted with licorice strokes. The dotted red lines indicate hydrogen bonds

**Fig. 6:**
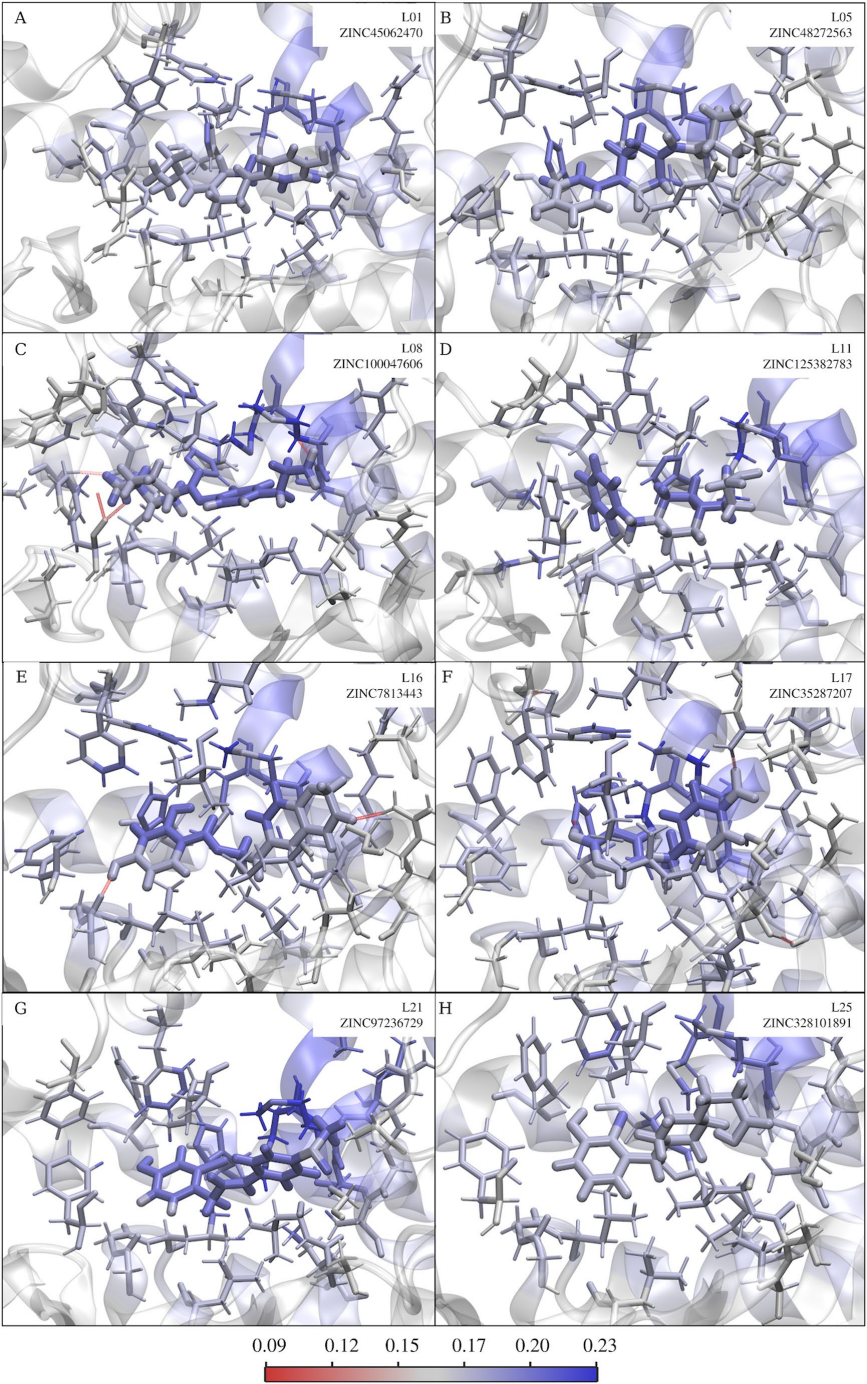
Representation of Fukui reactivity descriptor for TRα complexes. The reactivity index is colored with a red-white-blue color gradient, red being less reactive and blue being more reactive, as per the color legend. The ligands are highlighted with licorice strokes. The dotted red lines indicate hydrogen bonds

We calculated the TRα-ligand complexes using semiempirical quantum theory (PM7 MOZYME) and the Fukui reactivity descriptor to analyze the key interactions in protein-ligand complex formation. Subsequently, we visually represented the Fukui reactivity index with a color gradient (red-white-blue). The most reactive regions were found within the T3 binding cavity in the apo models, TRα-T3 complex (Fig. 5) and TRα-candidates complex (Fig. 6).

Considering the reference model of agonist activity, TRα-T3 (Fig. 5), the highest values of the Fukui index were observed within the binding cavity, particularly in the ligand and the nearby residues. In the ligand, the index is concentrated in the aromatic rings and the phenol group, while in the protein, the prominent residues are Arg262, Arg266, His381, Phe401, and Phe215. The elevated values are present in atoms involved in interactions between His381 and the phenol group of T3, between Phe401 and Phe215, and the aromatic rings of the ligand. Additionally, Arg262 and Arg266, to a lesser extent, interact with the carboxylic group of T3.

It is important to note that Fukui descriptors evaluate local reactivity, therefore, they mainly describe electrophilic and nucleophilic tendencies. These descriptors do not explicitly take into account non-covalent interactions, such as π–π stacking or dispersion-driven aromatic contributions, which may also play a role in stabilizing the protein-ligand. Thus, the present analysis focuses on electronic reactivity trends and does not fully capture all interaction components involved in binding, but they are very important from the point of view of evaluating key interactions from a qualitative standpoint (16, 17).

Evaluating the distribution of TRα amino acid residues revealed a predominance of reactive residues within 15 Å of the T3 coordinate center, which includes the binding cavity and its surroundings. In this region, arginine (Arg), asparagine (Asn), cysteine (Cys), phenylalanine (Phe) and threonine (Thr) are more prevalent, with an occurrence of 43%, 40%, 43%, 69% and 50%, respectively, in relation to the rest of the protein. Even Asn, Phe and Thr, non-ionizable residues, can participate in the charge-transfer process (47). The presence of the frontier orbitals and their neighborhood, up to 3 eV below the HOMO and above the LUMO, on the T3 atoms and at the interaction interface suggests that the electronic structure of the complex and the charge transfer may be associated with the molecular recognition, as a consequence of the hormone-receptor interaction.

Among the candidate ligands for TRα–T3 agonists, those that performed poorly in the QM/MM reranking (Fig. 4C) exhibited lower Fukui indices compared to the ligands that presented more favorable ΔH values, as observed in L01 and L25 (Fig. 6A and H). In contrast, the most successful molecules in the reranking, such as L08 and L17 (Fig. 6C and F), demonstrated a reactivity index close to the values calculated for the reference molecule, T3. Importantly, although some divergence was observed among binding energy estimation methods due to their different theoretical approximations, the DFT-based QM/MM approach was considered the most reliable for electronic interaction analysis. The agreement between QM/MM results and the Fukui reactivity descriptors strengthens the identification of L08 and L17 as promising TRα agonist candidates. These findings allow the identification of key interacting chemical groups and provide insights for rational structural modifications aimed at optimizing agonistic activity.

In general, we observed that the complexes formed with T3 and candidates (Figs. 5 and 6) show a Fukui reactivity pattern. Apparently, the reactivity is greater in the ligand and protein binding site in the complexes with T3 and candidate ligands that showed lower binding energy (∆G, ∆H and ∆E), Fig. 4. In contrast, the candidate molecules that showed higher interaction energy (less stable) have lower reactivity in the protein-ligand interaction region. This suggests that Fukui reactivity may be an important parameter for evaluating intermolecular interaction, especially candidate molecules for TRa agonists, which may extend to other molecular targets.

## Discussion

In the study developed by Wouters, McKee and Luyten (2020) (48), they presented estimates of costs for research and development of new drugs, using public information as a basis, showing that launching a single drug on the market may cost up to 2.8 billion dollars. To increase the efficiency of the prospection of new drugs, computational approaches are used (49). In a pioneering way Ferreira, Bartelt, Greene (1970) (50) used computational approaches for the development of Captopril, based on the structural knowledge of the potent inhibition of the Angiotensin Converting Enzyme by means of the peptide isolated from Bothrops jararaca (50), thus reducing the cost and time to make drugs available to the market.

Understanding the structure and dynamics of TRs is critical for appropriate choice of potential agonists. In high-throughput screening studies, Paul-Friedman et al. (2019) (51) explored the Tox21 environmental compound library, noting that the TR is a very selective receptor, providing limited chemical diversity of ligands with activity. In general, our most promising candidates showed similar characteristics with agonists described in the literature, featuring a hydrophobic body, with one end carried by a carboxylate and the other by a phenolic group.

The carboxylate is considered a fundamental component for conferring selectivity of T3 and analogues for TRs over other members of the nuclear receptor family. In the same vein, Dow et al. (2003) (34) concluded that the presence of phenol is critical for TR activation. However, Malm et al. (2007) (52) presented in his study several agonists that lacked the phenol group, yet had donors such as ketones and amines, with TRα agonist activity with similar potency to T3 in in vitro studies. Thus, L17 presents all the requirements discussed here. L08, on the other hand lacks phenol, but has an amino group, as well as phenol is a good hydrogen donor.

L08, selected at the end of the screening, is already a Japanese drug approved for commercial use as an anti-ulcer drug, called Cetraxate or Neuer^®^ (53). It is clinically safe, with no serious side effects during human studies by Ishimori, Yamagata and Taima (1979) (54). Also, in clinical toxicity trials, it has been used in daily doses of 800 mg for 8 weeks continuously (Lee et al., 1992) (55). Additionally, Cetraxate has other effects that may assist in cardiac injury events, such as those observed by Hashizume et al. (1979) (56), who reported inhibition of platelet aggregation and decreased vascular resistance. Effects that are currently obtained in the clinic, mainly using aspirin and beta blockers, indicated by the guidelines on treatment of acute myocardial infarction (57).

These considerations suggest good prospects for repositioning L08 (Cetraxate) for use in cardiac lesions involving loss of cardiomyocytes. Drug repositioning reduces the time and investment required for market insertion, being responsible for about 30% of new drugs and vaccines approved by the FDA in recent years (58). Such success is attributed mainly because the molecules are safe for use in humans, since about 90% of drug candidates fail in phase 1 clinical trials (59).

Importantly, another major challenge in the implementation of new drugs in the market is the chemical synthesis of the compounds and the expansion to industrial scale (60). This barrier can be remedied in the reuse of drugs, also, considering that Cetraxate has already been synthesized and intellectually protected by the company Pfizer Italy (1978) and the Japanese Aska Pharmaceutical, both implemented the synthesis on an industrial scale (61). Additionally, these patents have already been expired, opening the possibility for new clinical applications.

The fact that L08 is a commercial drug generates indications that L17 may present drug-like characteristics. This consideration is based on the principle that drug-like molecules share chemical characteristics (20). Such correlation has been refined since the study by Lipinski, Lombardo, Dominy, and Feeney (1997) (62) who developed a set of criteria, commonly known as the “rule of five.” Shultz (2018) (61) conducted a comprehensive review of FDA approved drugs over two decades since the Linpinskis article was published and demonstrated the influence of the “rule of five” and how these have been refined. This information assists during the process of new drug discovery, supporting the same principle of sharing chemical characteristics between drugs.

From a quantitative perspective, the integration of docking, molecular dynamics, and binding free energy calculations provides additional support for the selection of L08 and L17 as promising TRα agonist candidates. Both compounds exhibited stable binding modes throughout the MD simulations, with RMSD profiles indicating preservation of key interactions within the binding pocket. Furthermore, the MM/PBSA and QM/MM results suggest favorable interaction energies, consistent with the structural features required for TRα activation described in the literature.

In particular, L17 fulfills the canonical pharmacophoric requirements of TRα agonists, including the presence of both hydrogen donor and acceptor groups capable of interacting with residues such as His381 and Arg228, which are known to be critical for receptor activation. On the other hand, L08, despite lacking a phenolic group, maintains alternative hydrogen bonding capabilities, consistent with previous reports of non-classical agonists that retain activity through compensatory interactions. This observation highlights the ability of the proposed computational pipeline to identify both classical and non-classical agonists.

Thus the information obtained in silico, by various computational techniques, as well as literature review, served as a basis for better choice making during the virtual screening process in search of an alpha thyroid receptor agonist compound aimed at use in cardiac regeneration and proliferation, and two potentially active compounds were selected. One of the candidates is a drug approved for clinical use, allowing drug repositioning, and the other presents characteristics similar to drugs with potential innovation, because it has no described biological activity related to cardiac lesion.

## Conclusion

We performed a hierarchical virtual screening in search of potential thyroid receptor alpha agonist drugs for potential cardiac regeneration. Starting from the ZINC 15 database, filtering candidate molecules by physicochemical characteristics, Pharmacophore, Molecular Docking, Molecular Dynamics, Free Energy of Binding by MM/PBSA and Quantum Methods (PM7 and QMMM) and Fukui reactivity calculations, obtaining two potential ligands: L08 (ZINC100047606), Cetraxate (Neuer^®^) a drug used in the treatment of gastric ulcers; and L17 (ZINC35287207), compound with no biological activity described so far. We have shown insights into the greater reactivity in atoms that carry out interactions between the protein and the ligand can help in the process of optimizing drug candidate molecules.

## Supplementary Information

Below is the link to the electronic supplementary material.

Supplementary Material 1

## Data Availability

No datasets were generated or analysed during the current study.
