# Supplementary material for: Virtual screening of compounds for the development of thyroid hormone analogues for potential application in cardiac regeneration
Source: J Comput Aided Mol Des. 2026 Mar 23;40(1):81. doi: 10.1007/s10822-026-00787-5 (PMC13009001; doi:10.1007/s10822-026-00787-5)
Supplement: Supplementary file 1 — Supplementary Material 1 [file 10822_2026_787_MOESM1_ESM.docx]

**Supplementary Material**


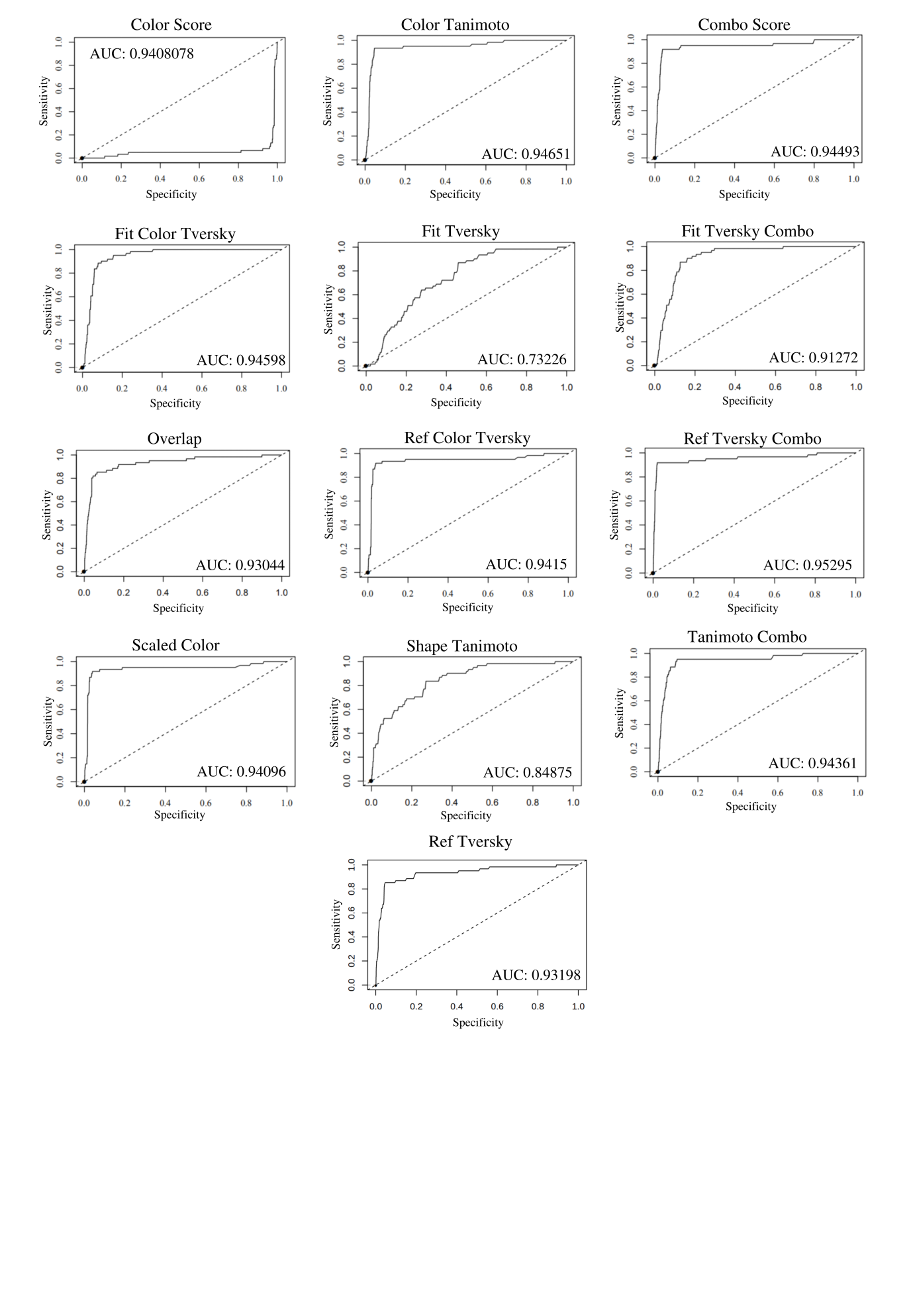


**Figure S1**. AUC of the ROCS scoring functions. On the x-axis is represented the sensitivity and on the y-axis the specificity for each ROCS scoring function for the pharmacophore model used in the virtual screening.

Ativos

Decoys


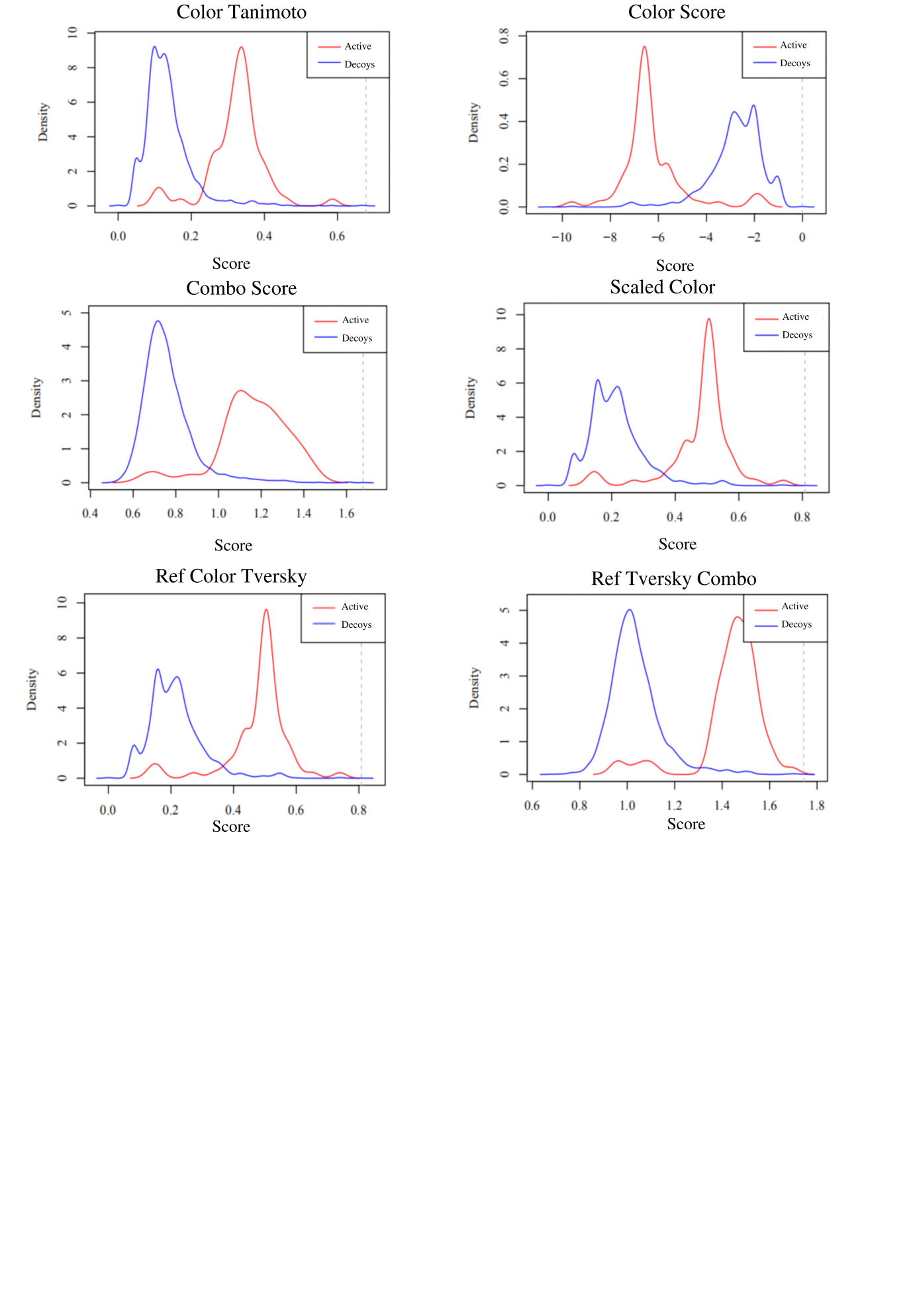


**Figure S2**. Scatter of populations of active and inactive molecules for TRα of the functions selected in Table 1. Red lines represent active ligands; blue lines represent inactive ligands. On the y-axis is represented the density of the populations, on the x-axis is shown the score of the populations.


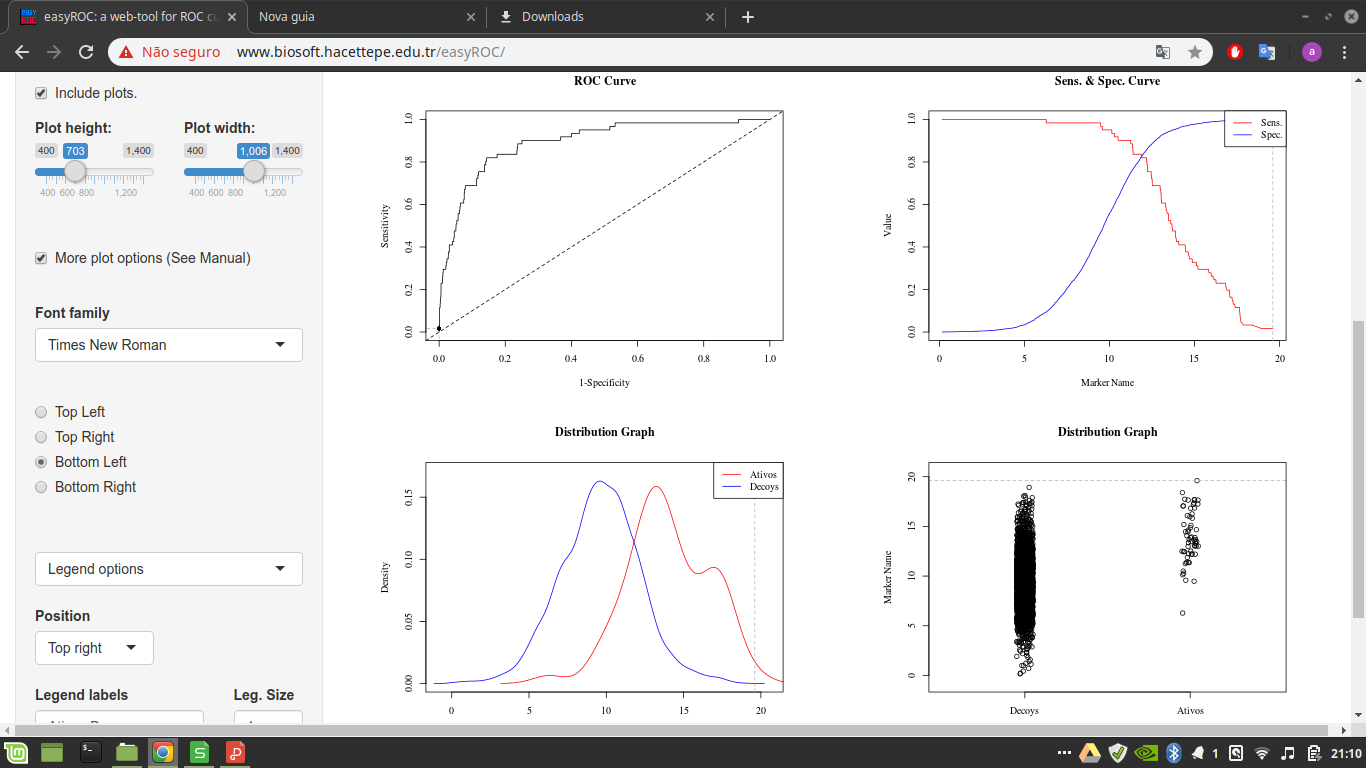


4lnw_oti

Specificity

Sensitivity

AUC: 0.8920536


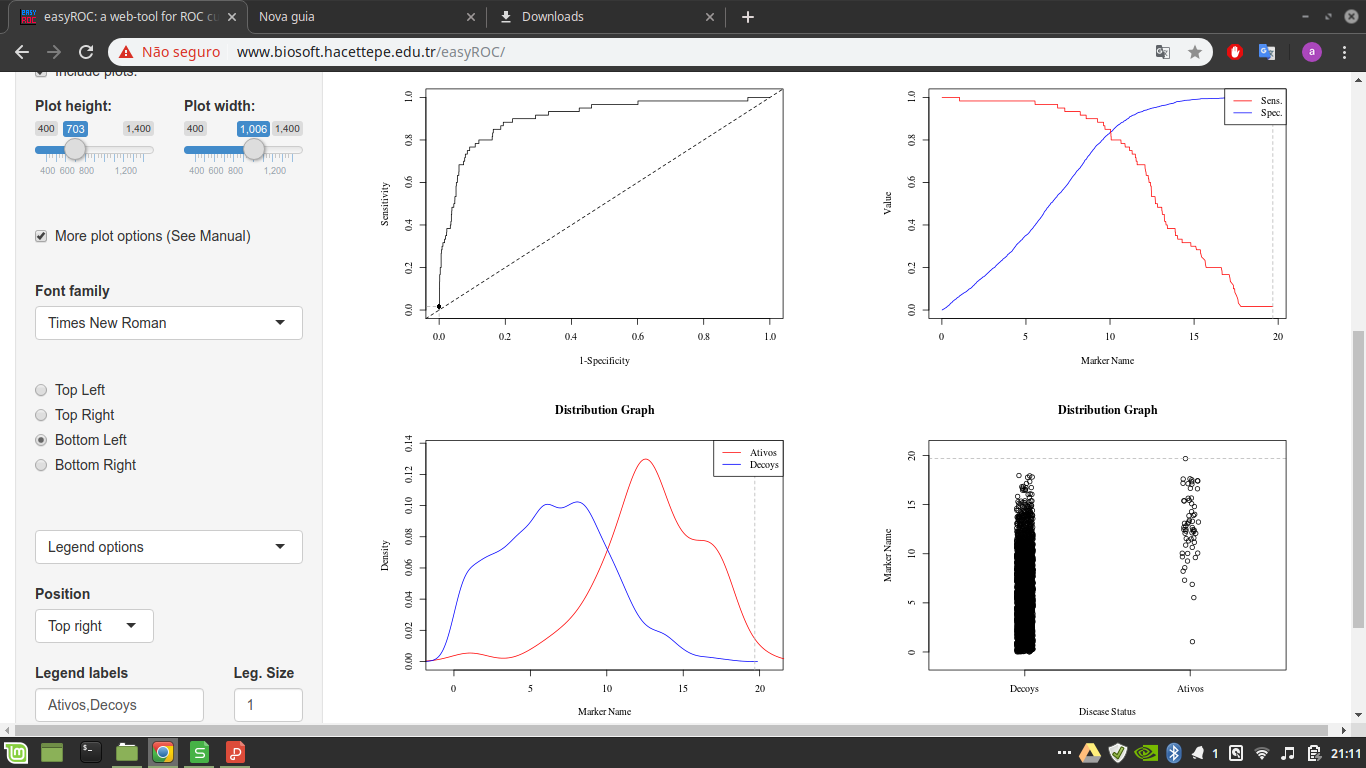


4lnw_arg228

Specificity

Sensitivity

AUC: 0.9037462


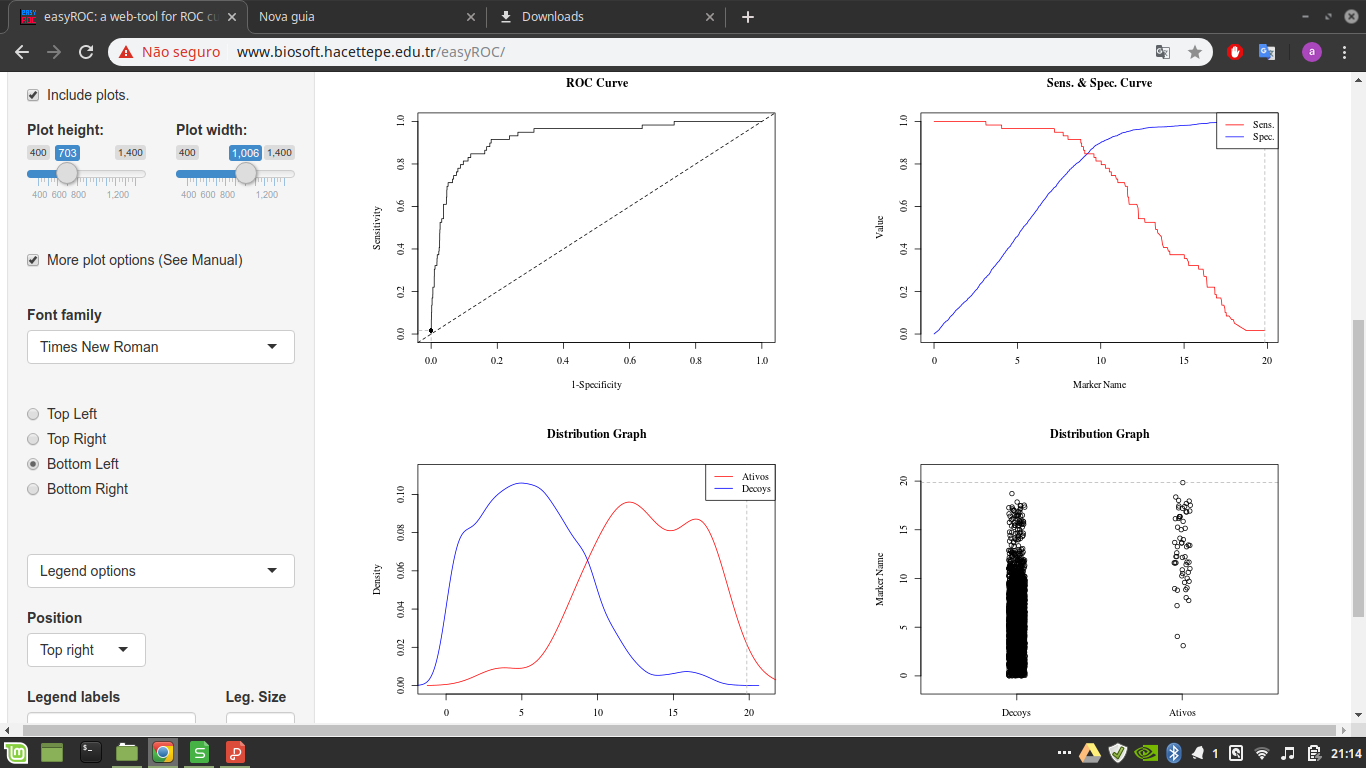


4lnw_his381

Specificity

Sensitivity

AUC: 0.9250974


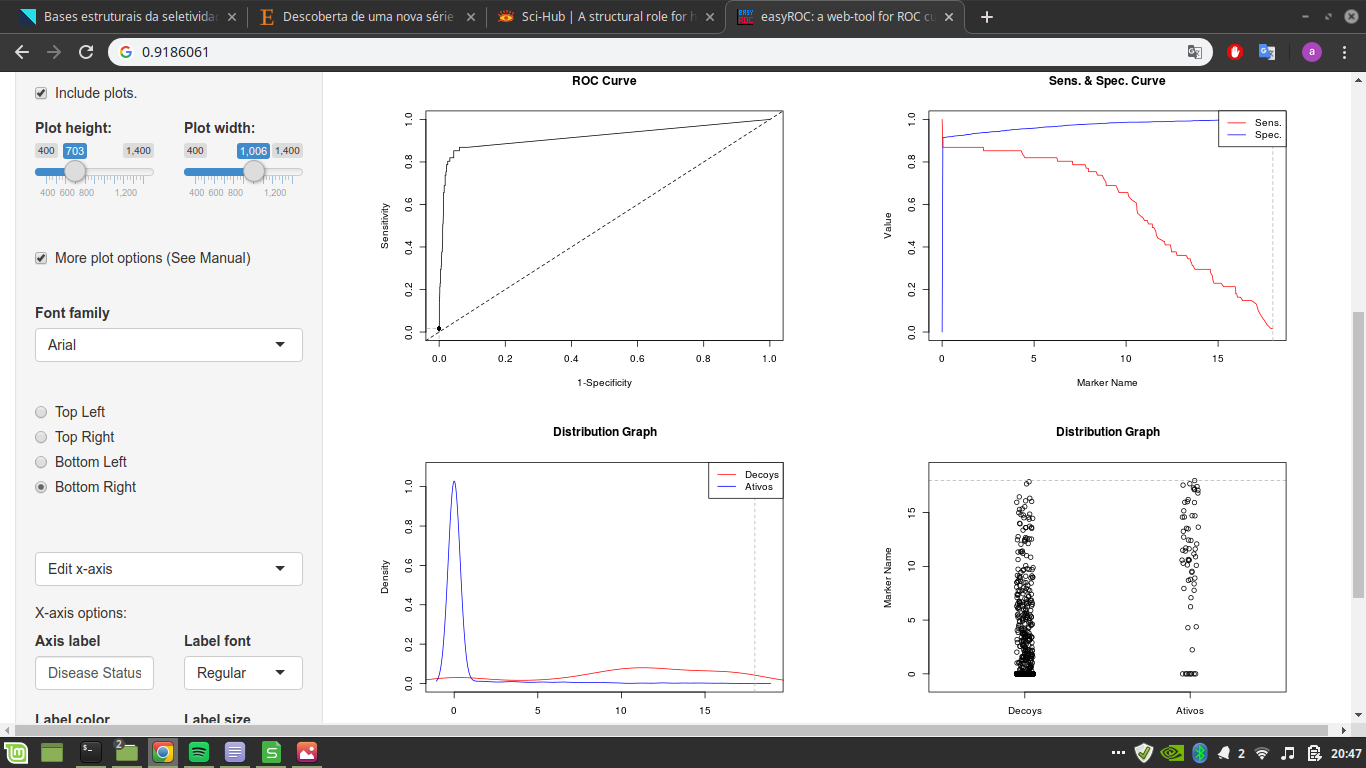


4lnw_his_arg

Specificity

Sensitivity

AUC: 0.9186061

**Figure S3.** AUC values with optimized molecular docking parameters.

**
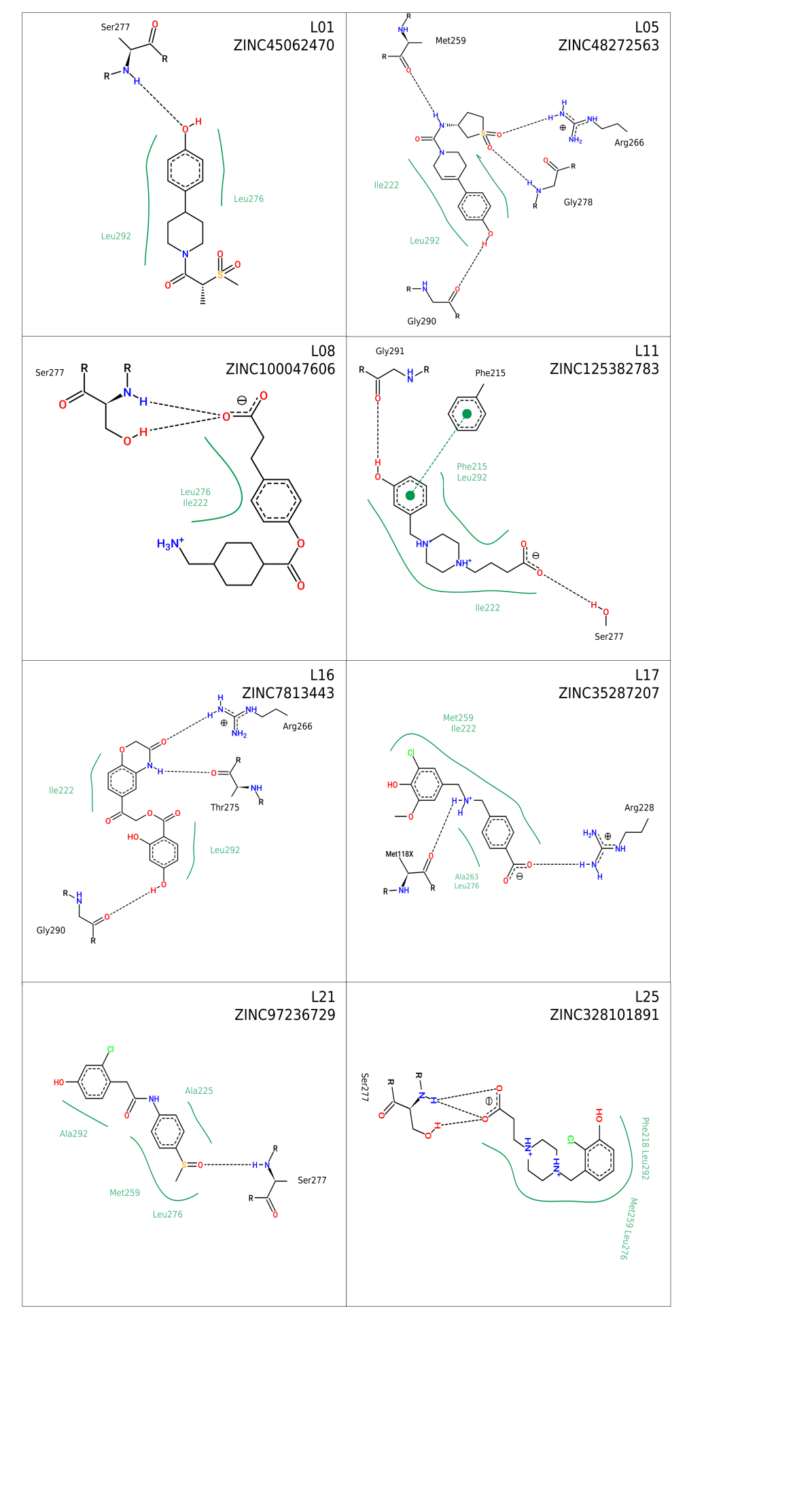
**

**Figure S4.** 2D representation of interactions performed during Molecular Dynamics.


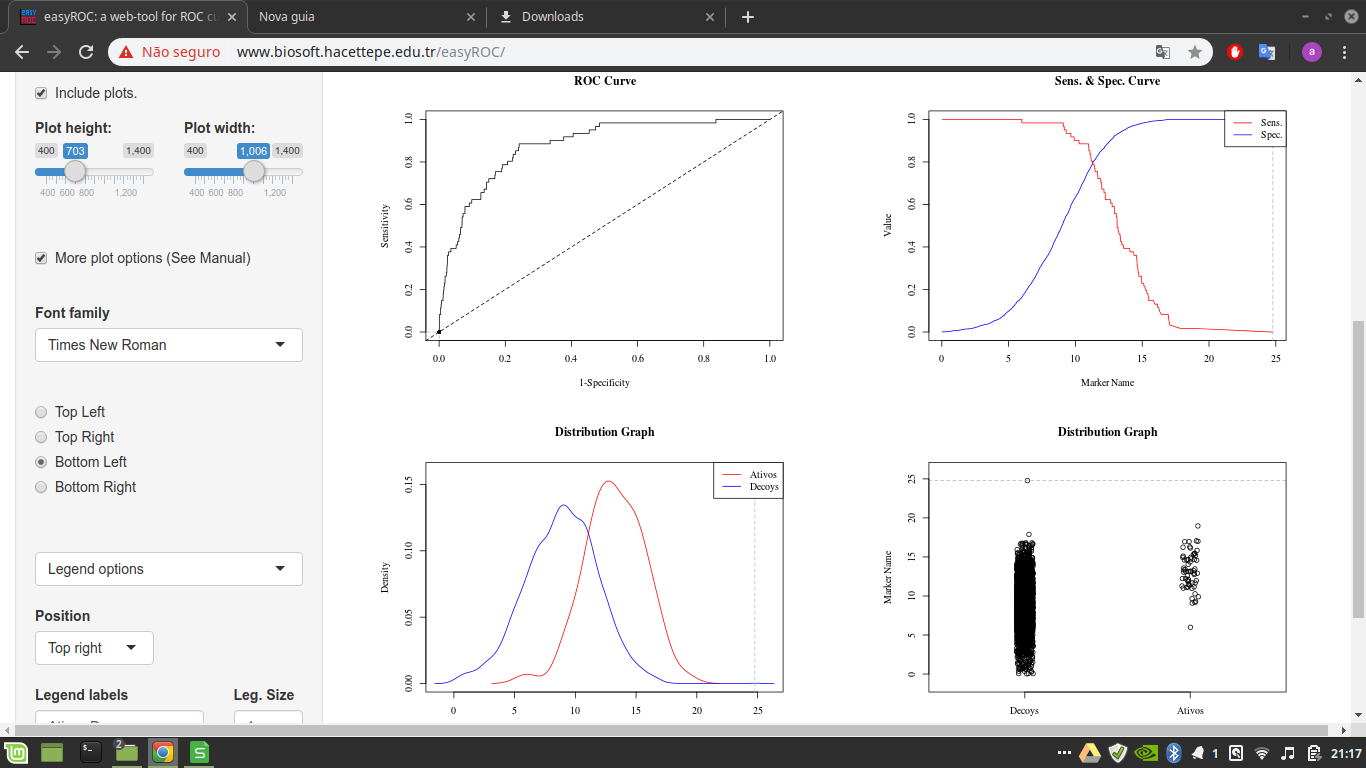


2h77

Specificity

Sensitivity

AUC: 0.8754312


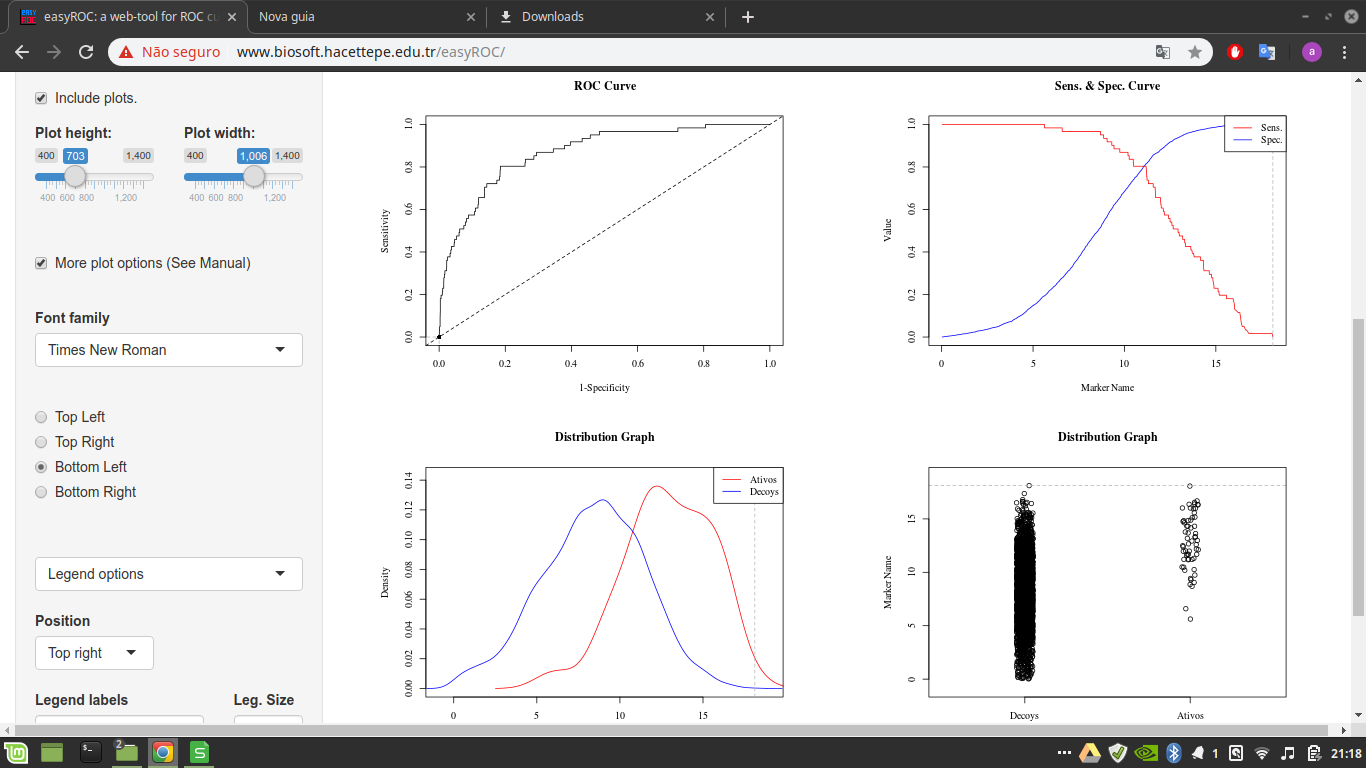


2h79

Specificity

Sensitivity

AUC: 0.867467


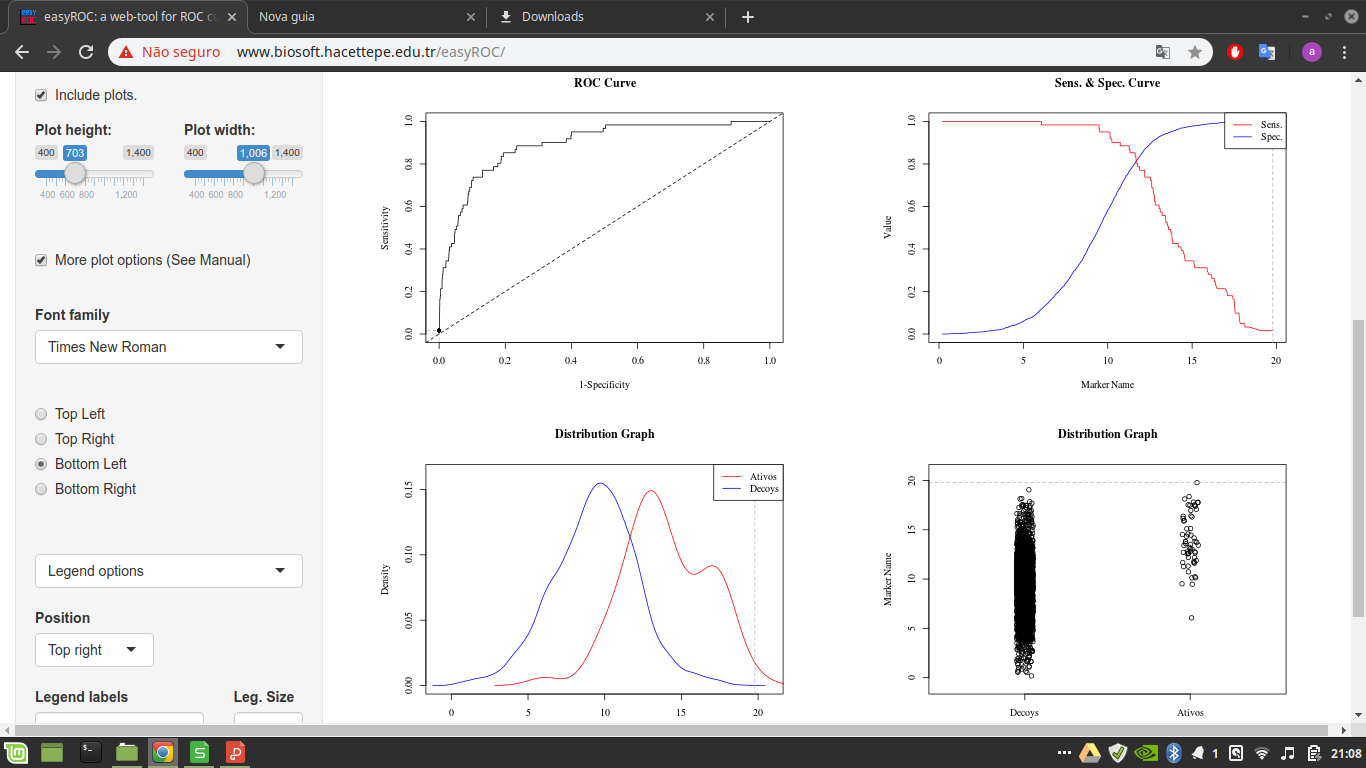


4lnw

Specificity

Sensitivity

AUC: 0.8908859


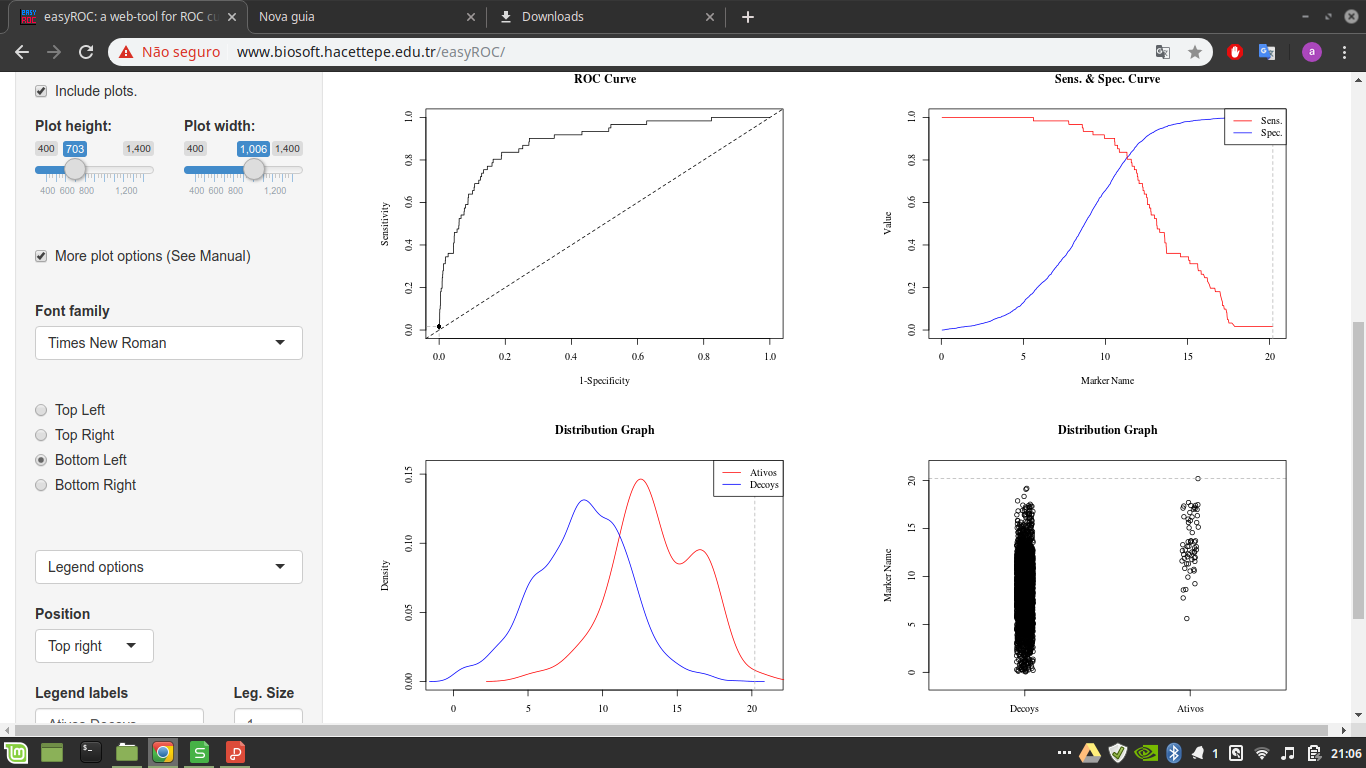


4lnx

Specificity

Sensitivity

AUC: 0.8818894

**Figure S5.** AUCs of crystallographic structures tested with molecular docking. Table S3 shows the RMSD values of the binding cavity residues, considering those at a distance of 5 Å from the ligand bound to TRα, obtained using the VMD program. Thus, we observe consensus between structures 4lnx, 2h77, and 2h79, with RMSD values less than 0.7 Å, which is sufficient to admit that they probably have similar performance in the molecular docking process. However, structure 4lnw has a disparity between the aforementioned structures, which could initially be a reason to dismiss it and use those that obtained consensus. However, the residue that causes this difference is Arg 228, which is fundamental for receptor activity, so it was decided to test all structures for validation with active and inactive ligands.


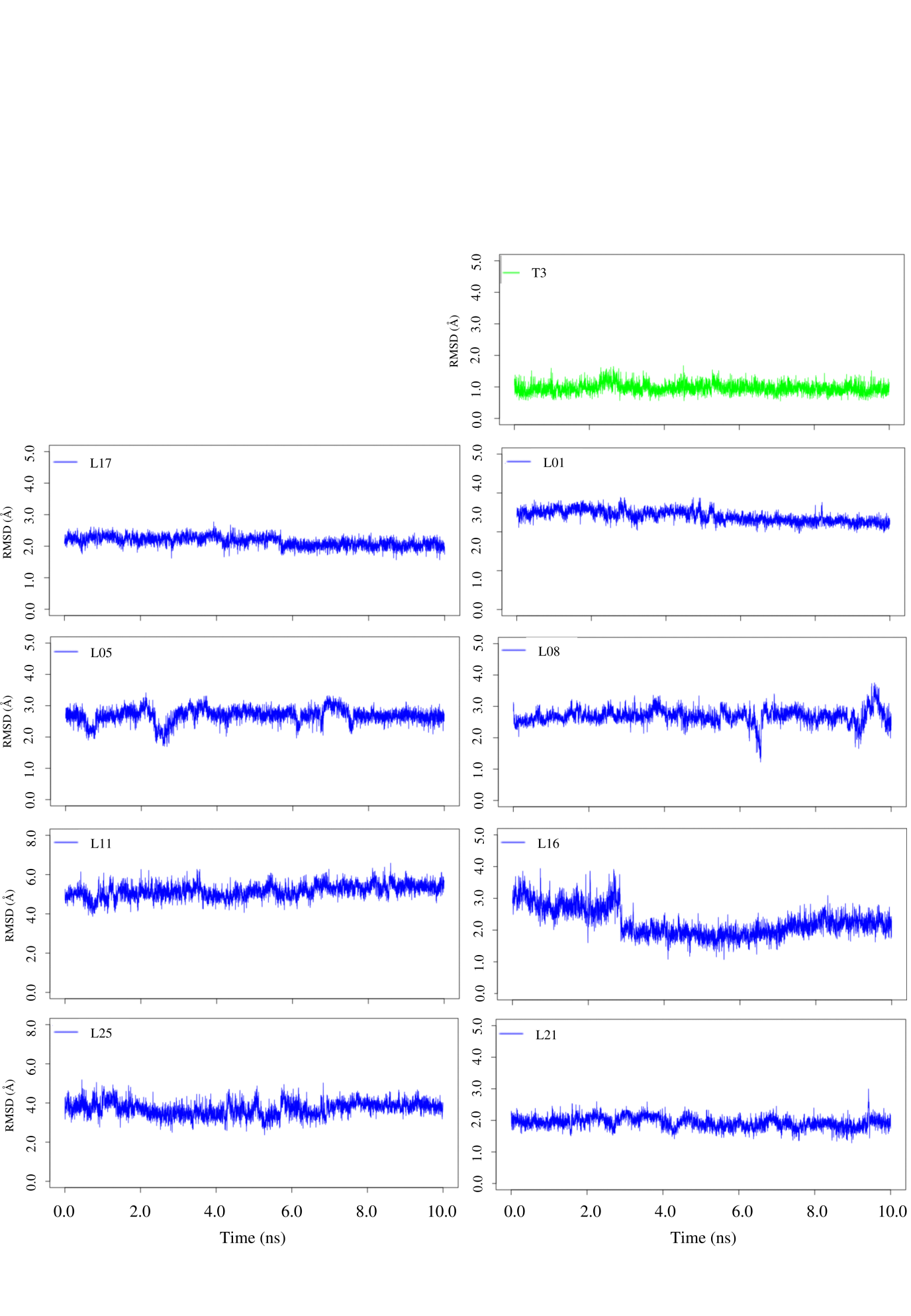


**Figure S6.** Root-mean-square deviation (RMSD) of ligand heavy atoms during 10 ns molecular dynamics simulations for the TRα–ligand complexes. The reference ligand (T3) is shown in green, while the candidate compounds (L01, L05, L08, L11, L16, L17, L21, and L25) are shown in blue. In general, the RMSD profiles indicate rapid stabilization within the first nanoseconds, followed by relatively stable fluctuations throughout the simulation time. Most ligands exhibit RMSD values in the range of ~2–3 Å, suggesting preservation of the binding mode within the receptor cavity. Some systems (e.g., L11 and L25) show higher fluctuations, which may indicate increased flexibility or partial rearrangement within the binding site. Overall, the results support that short MD simulations are sufficient to achieve local structural relaxation and stabilization of ligand binding poses for subsequent energy analyses.

| Ligand | ΔG_binding_ | ΔG_solv_ | ΔG_gás_ | EDISPER | ENPOLAR | EPB | EL | VDW |
| --- | --- | --- | --- | --- | --- | --- | --- | --- |
| T3 | -46.0253 (0.2053) | 36.1368 (0.2689) | -82.1621 (0.2988) | 62.5606 (0.0298) | -35.6289 (0.0172) | 9.2051 (0.2602) | -29.8363 (0.3295) | -52.3258 (0.1168) |
| L01 | -5.7648 (0.1392) | 63.8260 (0.1378) | -69.5908 (0.1322) | 55.7930 (0.0253) | -31.9614 (0.0170) | 39.9944 (0.1363) | -22.9089 (0.1362) | -46.6820 (0.0821) |
| L05 | 1.8108 (0.1540) | 85.3313 (0.1471) | -83.5205 (0.1144) | 59.4112 (0.0242) | -32.9506 (0.0197) | 58.8707 (0.1417) | -34.3164 (0.1149) | -49.2042 (0.0812) |
| L08 | -12.6900 (0.1763) | 96.6656 (0.1533) | -109.3556 (0.1717) | 58.1114 (0.0307) | -33.6614 (0.0172) | 72.2156 (0.1419) | -59.2146 (0.1772) | -50.1410 (0.0797) |
| L11 | -6.1911 (0.1445) | 61.9341 (0.1034) | -68.1252 (0.1177) | 53.9327 (0.0268) | -31.4667 (0.0143) | 39.4682 (0.0930) | -24.6552 (0.1238) | -43.4700 (0.0819) |
| L16 | 2.0862 (0.2082) | 78.4864 (0.1271) | -76.4001 (0.2127) | 58.5265 (0.0276) | -32.3282 (0.0227) | 52.2881 (0.1237) | -27.1726 (0.1877) | -49.2276 (0.0955) |
| L17 | -11.7210 (0.3617) | 154.4836 (0.2302) | -166.2046 (0.4763) | 54.4664 (0.0274) | -32.8982 (0.0125) | 132.9154 (0.2249) | -118.8258 (0.5158) | -47.3787 (0.1040) |
| L21 | 5.5733 (0.1631) | 73.8996 (0.1680) | -68.3262 (0.1761) | 54.6239 (0.0267) | -31.1072 (0.0213) | 50.3829 (0.1630) | -24.7273 (0.1822) | -43.5989 (0.0795) |
| L25 | -1.0887 (0.1364) | 59.1741 (0.1198) | -60.2628 (0.1119) | 52.0188 (0.0307) | -29.5262 (0.0200) | 36.6815 (0.1080) | -17.9014 (0.08480 | -42.3614 (0.0747) |

**Table S1.** Binding Free Energy and its components for the ligands. The table expresses the Free Binding Energy values for each ligand. The Standard Error of the Mean are provided in parentheses. In the columns are the Ligand; Binding Free Energy (ΔGLigation); Solvation Free Energy (ΔG_solv_ ); Vacuum Free Energy (ΔG_gás_ ); Dispersion Solvation Free Energy (ENDISPER); Polar Solvation Free Energy (ENPOLAR); Electrostatic Poisson-Boltzmann Solvation Contribution (EPB); Electrostatic Energy (EL); and Contribution from van der Waals interactions (VDW). All values are expressed in kcal/mol.

| **ChEMBL ID** | **Molecular Formula** |
| --- | --- |
| CHEMBL159682 | C25H24FNO6 |
| CHEMBL163228 | C20H17Br2N3O5 |
| CHEMBL191275 | C21H18O3 |
| CHEMBL2035874 | C18H17Br2NO5 |
| CHEMBL2035875 | C20H23NO5 |
| CHEMBL2035876 | C18H16Cl3NO5 |
| CHEMBL2035877 | C21H25NO5 |
| CHEMBL2035878 | C22H25NO5 |
| CHEMBL2035879 | C21H22BrNO5 |
| CHEMBL2035880 | C22H24BrNO5 |
| CHEMBL2035881 | C21H23NO5 |
| CHEMBL2035882 | C22H25NO5 |
| CHEMBL2035883 | C21H25NO6S |
| CHEMBL2035884 | C23H27NO4 |
| CHEMBL2035885 | C23H25NO5 |
| CHEMBL217221 | C25H23Br2NO4 |
| CHEMBL2312262 | C24H23N3O6 |
| CHEMBL2312263 | C27H27NO5 |
| CHEMBL2312264 | C27H26FNO5 |
| CHEMBL2312265 | C27H27NO6 |
| CHEMBL2312266 | C27H25NO6 |
| CHEMBL2312267 | C28H29NO5 |
| CHEMBL2312268 | C27H25F2NO5 |
| CHEMBL2312269 | C27H25F2NO5 |
| CHEMBL2312270 | C27H26ClNO5 |
| CHEMBL2312271 | C27H26ClNO5 |
| CHEMBL2312272 | C27H26ClNO5 |
| CHEMBL2312273 | C27H27NO6 |
| CHEMBL2312274 | C27H27NO6 |
| CHEMBL2312275 | C27H27NO6 |
| CHEMBL2312276 | C19H19NO5 |
| CHEMBL2312277 | C21H23NO5 |
| CHEMBL2312278 | C23H27NO5 |
| CHEMBL2312279 | C24H29NO5 |
| CHEMBL2312280 | C27H33NO5 |
| CHEMBL2312281 | C26H24FNO5 |
| CHEMBL243207 | C22H26N2O3S |
| CHEMBL243415 | C27H36N2O3S |
| CHEMBL243416 | C28H30N2O3S |
| CHEMBL243417 | C25H32N2O3S |
| CHEMBL244211 | C26H32N2O3S |
| CHEMBL456237 | C21H12ClF5N2 |
| CHEMBL457977 | C21H13F5N2 |
| CHEMBL470930 | C21H18Cl2N2O7S |
| CHEMBL471780 | C20H16Cl2N2O8S |
| CHEMBL479583 | C27H24Cl2N2O6 |
| CHEMBL479584 | C19H14Cl2N2O6 |
| CHEMBL480119 | C16H9Cl2NO5 |
| CHEMBL480124 | C23H20Cl2N2O6 |
| CHEMBL480132 | C24H20Cl2N2O6 |
| CHEMBL480312 | C19H15Cl2NO5 |
| CHEMBL480313 | C20H17Cl2NO5 |
| CHEMBL480916 | C21H16Cl2N2O7 |
| CHEMBL480917 | C22H20Cl2N2O7S |
| CHEMBL481487 | C21H16Cl2N2O6 |
| CHEMBL481673 | C22H18Cl2N2O6 |
| CHEMBL482251 | C23H15Cl2NO5 |
| CHEMBL506944 | C20H16Cl2N2O6 |
| CHEMBL520119 | C22H13Cl2NO6 |
| CHEMBL522936 | C21H18O3 |
| CHEMBL552240 | C28H23ClF3NO5 |

**Table S2.** Agonist molecules for the thyroid hormone receptor alpha. The molecules were obtained using ChEMBL as a base by searching for the term "Thyroid hormone receptor alpha", targeted with code CHEMBL1860, selected only with agonist activity.

| PDB ID | RMSD |
| --- | --- |
| 2h77 | Ref. |
| 2h79 | 0.6934 |
| 4lnx | 0.6964 |
| 4lnw | 5.3734 |

**Table S3.** RMSD of structures linked to T3 of TRα. RMSD: Root mean square deviation. RMSD values were approximated. Ref.: Structure used as a reference for RMSD calculations.

| Ligand | Fukui (a.u.) |
| --- | --- |
| T3 | 6.582299 |
| L01 | 8.076965 |
| L05 | 8.351027 |
| L08 | 9.082188 |
| L11 | 8.351939 |
| L16 | 7.436170 |
| L17 | 7.611846 |
| L21 | 7.291429 |
| L25 | 7.463125 |

**Table S4.** Fukui reactivity descriptor values for T3 and candidate ligands.
